# The malate sensing two-component system MaeKR is a non-canonical class of sensory complex for C4-dicarboxylates

**DOI:** 10.1038/s41598-017-02900-z

**Published:** 2017-06-02

**Authors:** L. Miguel-Romero, P. Casino, J. M. Landete, V. Monedero, M. Zúñiga, A. Marina

**Affiliations:** 1 Department of Genomic and Proteomic, Instituto de Biomedicina de Valencia (IBV-CSIC), Jaume Roig 11, 46010 Valencia, Spain; 2 Departamento de Bioquímica, Universitat de València, Dr Moliner 50, 46100 Burjassot, Spain; 3 Estructura de Recerca Interdisciplinar en Biotecnologia i Biomedicina (ERI BIOTECMED), Universitat de València, Dr Moliner 50, 46100 Burjassot, Spain; 4 Departamento de Biotecnología de Alimentos, Instituto de Agroquímica y Tecnología de Alimentos (IATA-CSIC), Av. Agustín Escardino 7, 46980 Paterna, Valencia Spain; 5 Group 739 of the Centro de Investigación Biomédica en Red sobre Enfermedades Raras (CIBERER) del Instituto de Salud Carlos III, -, Spain; 6 Departamento de Tecnología de Alimentos, Instituto Nacional de Investigación y Tecnología Agraria y Alimentaria (INIA), Carretera de La Coruña Km 7.5, 28040 Madrid, Spain

## Abstract

Microbial colonization of different environments is enabled to a great extent by the plasticity of their sensory mechanisms, among them, the two-component signal transduction systems (TCS). Here, an example of TCS plasticity is presented: the regulation of L-malate catabolism via malic enzyme by MaeRK in *Lactobacillales*. MaeKR belongs to the citrate family of TCS as the *Escherichia coli* DcuSR system. We show that the *Lactobacillus casei* histidine-kinase MaeK is defective in autophosphorylation activity as it lacks a functional catalytic and ATP binding domain. The cognate response regulator MaeR was poorly phosphorylated at its phosphoacceptor Asp *in vitro*. This phosphorylation, however, enhanced MaeR binding *in vitro* to its target sites and it was required for induction of regulated genes *in vivo*. Elucidation of the MaeR structure revealed that response regulator dimerization is accomplished by the swapping of α4-β5-α5 elements between two monomers, generating a phosphoacceptor competent conformation. Sequence and phylogenetic analyses showed that the MaeKR peculiarities are not exclusive to *L. casei* as they are shared by the rest of orthologous systems of *Lactobacillales*. Our results reveal MaeKR as a non-canonical TCS displaying distinctive features: a swapped response regulator and a sensor histidine kinase lacking ATP-dependent kinase activity.

## Introduction

To sense and to respond to environmental changes and stress conditions, all living organisms have developed different signal transduction systems. In microorganisms, two-component systems (TCS) represent a major signaling mechanism. A prototypical TCS consists of a sensor histidine kinase (HK) and an effector response regulator (RR). Signal transduction by canonical TCSs is known to occur basically by a mechanism of three reactions where, upon signal detection, the HK is autophosphorylated in a conserved His residue^1, 2^. Subsequently, the phosphoryl group is transferred to a conserved Asp residue on a cognate RR. This phosphorylation induces conformational changes in the RR that modulate its activity, generally DNA binding, since most RRs are transcriptional factors. Signal transduction is terminated upon RR dephosphorylation, mediated by endogenous phosphatase activity of its cognate HK, action of auxiliary phosphatases or by RR auto-hydrolysis^3–5^. Since TCS must discriminate and respond to specific stimuli among a vast diversity of signals generating quite diverse responses, the sensor domains in the HKs and the effector domains in the RRs present a high variability in sequence and structure. Contrarily, the catalytic domains involved in the autophosphorylation, phosphotransfer and phosphatase reactions are highly conserved both in HKs and RRs^6^. The catalytic core of HKs consists of two domains. The first one, the dimerization and histidine-phosphotransfer (DHp) domain, holds the phosphorylatable His and mediates HK dimerization. This domain is folded as two long antiparallel helices connected by a variable loop that forms a stable four-helix bundle in the HK homodimer^5, 6^. The second conserved HK domain, named CA (catalytic and ATP binding), binds the ATP molecule and catalyzes the phosphorylation of the histidine in the DHp domain. CA domains present a Bergerat or GHKL fold shared with other ATP-binding proteins (gyrases, Hsp90, MutL). The structure is an α/β sandwich consisting of a five-stranded mixed β sheet and three α helices in HKs^5, 7^. On the other hand, the RR presents a conserved receiver (REC) domain containing the phosphoacceptor Asp. The REC domain is folded with a (βα)_5_ topology where a central five-stranded parallel β-sheet is surrounded by three α-helices in one side and two in the other^8^. REC domains present the molecular machinery to recognize and catalyse the phosphoryl group transfer from the cognate HK. Asp phosphorylation of the RR REC domain triggers a change in the rotameric state of highly conserved Thr/Ser and Tyr/Phe residues in β4 and β5, respectively, inducing the conformational changes observed between the active and inactive states^8, 9^. This activation mechanism, named Y-T switch, seems to be predominant among RRs although alternative activation modes have been described for some RR subfamilies such as NtrC^9, 10^.

As our knowledge about TCS increases, variations of this basic signalling paradigm, including new regulatory steps, are described. For example, ancillary proteins can regulate any of the three signalling reactions or work as co-sensors together with the HK, or unphosphorylated RRs can also play a critical role in the regulation of gene expression^11–15^. In this way, the combination of a highly conserved catalytic machinery with variable sensor and effector domains together with the participation of ancillary proteins, provide TCSs of an extremely high signalling plasticity allowing these systems to generate species-specific responses to similar stimulus.

One example would be the regulation of C4-dicarboxylates uptake, which in *Escherichia coli* involves the TCS DcuS/DcuR and the secondary transporters DctA or DcuB^16^. In lactic acid bacteria (LAB), the metabolism of the C4-dicarboxylate L-malate takes place via two distinct pathways. One allows the conversion of L-malate into L-lactate via the function of the malolactic enzyme (MLE) pathway and the other converts L-malate to pyruvate via the malic enzyme (ME). *Lactobacillus casei* is a facultatively heterofermentative LAB frequently used as a starter culture for the production of fermented food products. *L. casei* is remarkable among LAB since it is one of the few species with the ability to utilize L-malic acid via the ME or the MLE pathways^17–19^. Regulation of the transcription of the genes involved in the utilization of L-malate via the ME pathway is under control of a TCS of the citrate family^18^, whereas the MLE pathway is under control of a LysR-type transcriptional regulator^19^. Although both pathways utilize the same substrate, the transcription of the corresponding genes is independently regulated^19^.

Genes involved in the ME pathway are arranged in two diverging operons, *maePE* and *maeKR*, encoding a putative L-malate transporter (*maeP*), an ME (*maeE*), and a TCS (*maeK* and *maeR*). This cluster is only found in a few species of the *Lactobacillales* order^18, 20^. It has been shown that ME is required for growth with L-malate and that both *maeK* and *maeR* are essential for expression of *maePE* ^18^. Requirement of *maeR* for *maePE* expression has been also demonstrated in *Enterococcus faecalis* JH2-2^21^ and requirement of *maeK* has been demonstrated in *Streptococcus pyogenes* ^22^. Transcriptional analyses showed that the expression of the *maePE* operon was induced by L-malate and repressed by glucose in *L. casei* ^18, 19^ and *E. faecalis* ^21^. In *S. pyogenes* and *L. casei*, in addition to L-malate, low pH also induced the expression of *maePE* ^22^. Interestingly, it has been shown that induction by low pH is independent of L-malate but requires a functional MaeK in *S. pyogenes* ^22^.

As noted above, induction by L-malate was dependent on a functional MaeKR TCS. Furthermore, inactivation of the genes encoding L-malate transporters in *L. casei* (*maeP* and *mleT*; the latter associated to the malolactic gene cluster) did not affect the transcription of *maeE* in the presence of L-malate^19^. This result, together with the absence of genes encoding putative solute binding proteins, suggests that no co-sensor proteins are participating in the regulation of MaeK activity, in contrast to other TCS of the citrate family (e.g. DcuS)^23^.

In order to understand these differences in the mechanism of regulation by MaeKR of *L. casei*, we sought to characterize the activity of both proteins *in vitro* and *in vivo*. We showed that MaeK forms a particular group within the HKs, characterized by a lack of a functional ATP-binding domain. We also solved the crystal structure of the REC domain of MaeR in its activated state as well as the structure of a mutant version defective in the phosphoacceptor Asp. Finally, we characterized the interaction of MaeR with the *mae* promoter region. Altogether, our analysis has revealed that MaeKR is a non-canonical TCS conserved in *Lactobacillales* and that the structure of MaeR acknowledges the α4β5α5-swapped dimers as a new class of dimerization for RRs.

## Results

### *L. casei* MaeK lacks the essential conserved motifs for ATP binding

**Figure 1:**
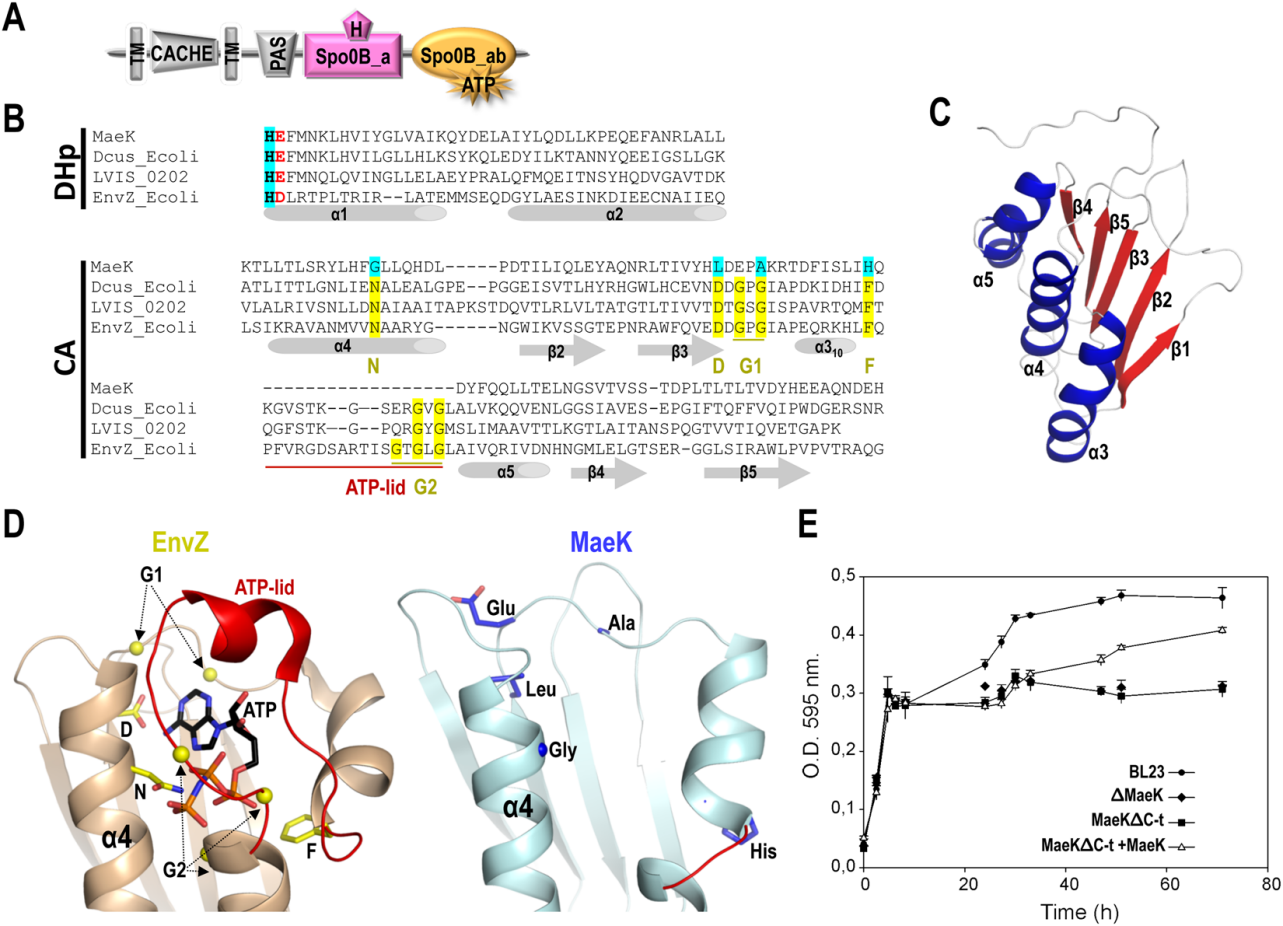
Domain composition and structural description of MaeK. (**A**) Graphical representation of the domains contained in MaeK, obtained with the SMART database and Pfam. TM correspond to transmembrane helix. (**B**) Sequence alignment of the Spo0B_a domain of MaeK with DHp domain of DcuS and EnvZ of *E. coli* containing the phosphorylatable His (in bold with blue pattern) and the subsequent catalytic acidic residue (colored in red). Below is represented the sequence alignment of the Spo0B_ab domain (degenerated CA domain) of MaeK with the CA domain of CitB from *Lactobacillus brevis* (LVIS_0202), and DcuS (DcuS_Ecoli) and EnvZ (EnvZ_Ecoli) from *E. coli* highlighting the conserved boxes for a canonical CA domain with their conserved specific residues in yellow pattern. Absence of conserved specific residues in MaeK contain blue pattern. (**C**) 3D structural model for the degenerated CA domain of MaeK generated by the program I-Tasser (helices in blue and beta strands in red). (**D**) Superposed 3D CA domain structures for EnvZ and MaeK to highlight the absence of conserved boxes and ATP-lid in MaeK with respect to EnvZ (colored as in B). (**E**) Growth of wild-type *L. casei* (BL23) and MaeK derivative strains. Wild-type (BL23), MaeK gene defective (∆MaeK), MaeK lacking CA domain (MaeKΔC-t) and MaeKΔC-t complemented with a plasmid expressing MaeK (MaeKΔC-t + MaeK) strains were grown in MEI medium supplemented with 5 g liter^−1^ of L-malic acid. Values represent the means of three independent experiments; error bars represent standard deviations.

In order to dissect the functional mechanism of MaeKR, we analysed the domain architecture of both proteins using Pfam and SMART servers. The search revealed that the sensor MaeK presents two transmembrane helices connected through an extracellular sensor domain consisting of a single Cache domain type 3 (PF17203), followed by a cytoplasmic PAS sensor domain (CL0183; residues 212–285), an alpha-helical domain SPOB_a (PF14689; residues 308–375) and a phospho-transferase B, C-terminal SPOB_ab domain (PF14682; residues 383–514) (Fig. 1A). However, and surprisingly for a putative sensor HK, the analysis did not reveal the presence of DHp phosphoacceptor (PFAM: HisKA or HisKA_3) or CA ATP-binding (PFAM: HATPase_c) domains, the canonical HK catalytic domains. Further comparative sequence analysis with canonical HKs allowed us to identify a putative phosphorylatable histidine (His334) in the SPOB_a helical domain, which corresponds to the phosphoacceptor His in Spo0B-type phosphotransferases (Fig. 1B). In addition, this His is followed by a catalytic acidic residue (Glu335) as is observed in canonical HKs^2^ (Fig. 1B), further supporting the hypothesis that this domain (residues 308–375) is a functional DHp domain. Contrarily, the sequence analysis of the C-terminal region of MaeK revealed the absence of the conserved motifs or “boxes” characteristics of HK CA domains necessary for ATP binding (Fig. 1B). These boxes provide key residues for the ATP-binding pocket and give specificity for this nucleotide. The N box is named after a conserved Asn that coordinates the magnesium ion for catalysis, D box after an Asp that gives specificity for adenine, the G boxes after two Gly rich regions that confer flexibility to the characteristic loop covering the ATP pocket (ATP-lid) and the F box after a Phe positioned at the beginning of the ATP-lid^7^ (Fig. 1B). To further confirm the absence of the canonical ATP-binding pocket in this domain and in order to avoid a possible misalignment of the sequence, we generated a structural model of the C-terminal domain of MaeK, using the I-TASSER server^24^. The model presents the characteristic architecture of HK CA domains with five β strands and three α helices forming an αβ sandwich (Fig. 1C). Superposition with the CA domain of the prototypical HK EnvZ (PDB:4KP4), confirmed the absence in MaeK of the conserved boxes essential for ATP binding, as well as the characteristic ATP-lid (Fig. 1D). Furthermore, docking an ATP molecule in the modelled putative CA domain of MaeK, according to the structure of EnvZ bound to AMPPNP (non-hydrolysable ATP analogue; PDB 4KP4), showed that the nucleotide accommodation would be sterically precluded by the presence of residues that occupies the binding pocket (Supplementary Fig. 1).

In order to check, *in vitro*, the autophosphorylation activity of MaeK we produced the recombinant complete cytoplasmic portion of MaeK (MaeK_C_). The protein eluted in gel filtration chromatography as a single peak corresponding to the expected size of the dimer species (Supplementary Fig. 1), a quaternary structure observed in functional HKs^25^. Furthermore, analysis of the circular dichroism spectra for MaeK_C_ estimated a secondary structure composition of 32% α-helix and 17.8% β-strand which correlated with the secondary structure composition predicted from the *in silico* model (37% α-helix and 22% β-strand) (Supplementary Fig. 1), supporting that MaeK_C_ is well-folded. We checked *in vitro* the kinase capacity of MaeK_C_ using [γ-^32^P]ATP as substrate, and no autophosphorylation was observed, even at high concentrations of radiolabeled ATP, long incubation times or in the presence of different divalent ions, conditions where the corresponding portions of canonical HKs showed autophosphorylation (Supplementary Fig. 2). We also measured by isothermal calorimetry (ITC) the binding affinity of MaeK_C_ for AMPPNP in the presence of 10 mM MgCl_2_, a divalent cation required for catalysis (Supplementary Fig. 2). Kd constant values higher than 1 mM were obtained, which reflects non-binding in the ITC system used, either for AMPPNP-MgCl_2_ at high concentrations or MgCl_2_ alone (Supplementary Fig. 2). Parallel assays with HK853, a canonical HK with autophosphorylation capacity, were carried out to validate the assay (Supplementary Fig. 2). This result confirms that MaeK_C_ is unable to bind nucleotide or metal ion even at physiological concentrations. Altogether, the *in silico* and *in vitro* analysis of MaeK strongly suggest that, even though it conserves a degenerated CA domain, it has lost the capacity to bind ATP and consequently, its putative autophosphorylation activity. However, MaeK seems to maintain an active phosphorylatable His, suggesting that it might conserve phosphotransferase and/or phosphatase activities. In fact, MaeK contains the E/DxxT/N motif, after the catalytic His, which has been demonstrated to be critical for the phosphatase activity in sensors that dephosphorylate receiver domains^26^ (Fig. 1B).

### Deletion of the degenerated CA domain of *L. casei* MaeK prevents growth in malic acid

We had previously observed that a complete deletion of *maeK* (Δ*maeK*) in *L. casei* led to an absence of growth in medium containing L-malate as a carbon source, and this deficiency was totally overcome by expression of MaeK from a plasmid^18^. Here we sought to analyse whether the C-terminal domain of MaeK, which is devoid of all the characteristics of an ATP-binding domain and, consequently, kinase activity, was necessary for activation of the *mae* promoter. To this end, a *L. casei* strain (MaeKΔC-t) expressing a MaeK variant lacking the C-terminal domain (residues 378–514) was obtained. As previously observed with the Δ*maeK* strain, MaeKΔC-t strain could not grow on L-malate (Fig. 1E). This observation suggests that the deleted domain is necessary for MaeR activation, although an incorrect membrane insertion/folding of MaeKΔC-t cannot be excluded. In contrast to the results obtained with the Δ*maeK* strain, complementation of MaeKΔC-t with a plasmid expressing MaeK only showed partial restoration of growth on L-malate (Fig. 1E). The partial restoration of growth observed in this strain could be explained by the formation of MaeK/MaeKΔC-t heterodimers with reduced activity compared with wild-type homodimers.

### The *L. caseimae* homologous genes share a common evolutionary history in Lactobacillales

**Figure 2:**
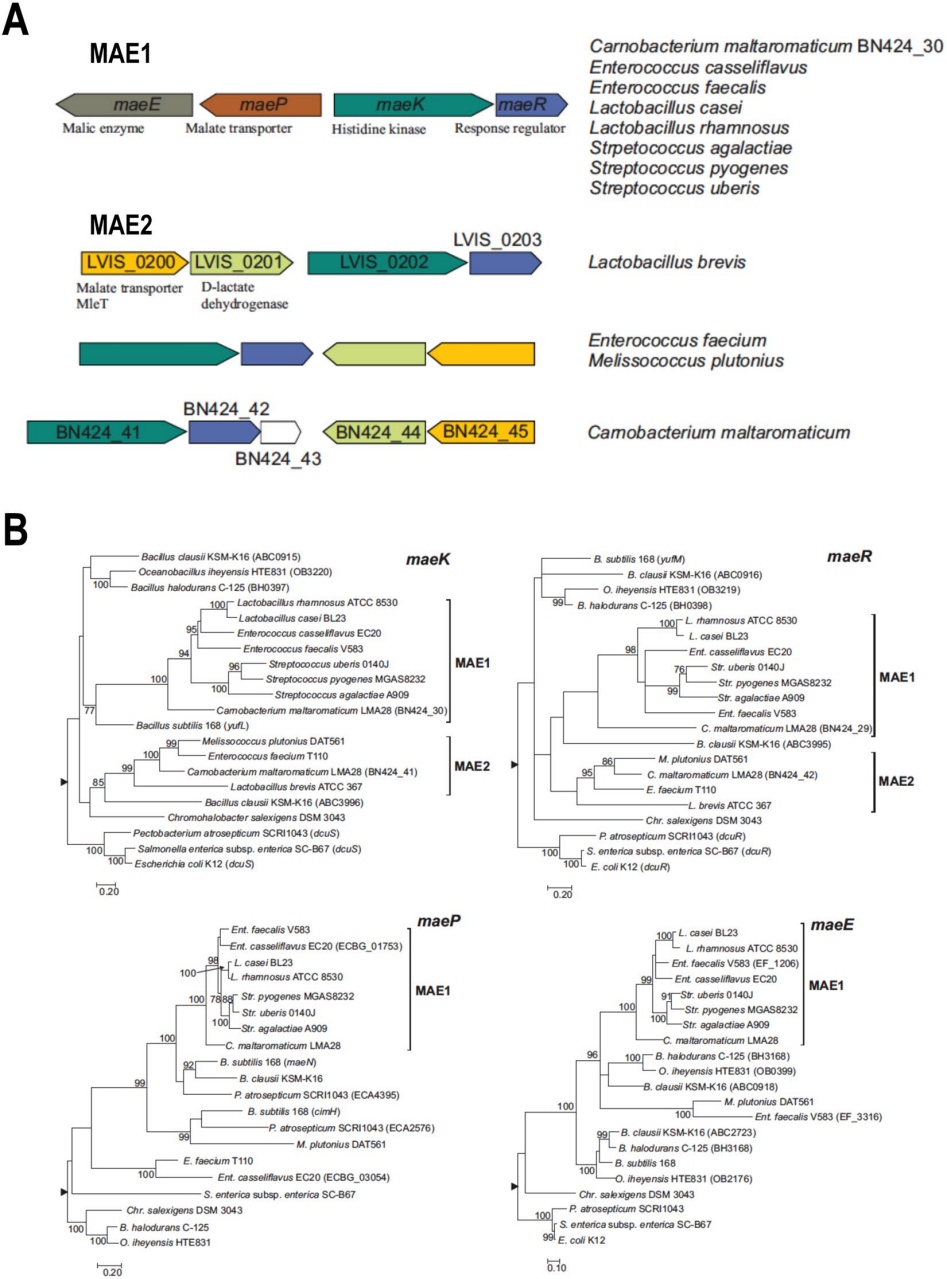
Clusters of *mae* genes and phylogenetic analysis. (**A**) Arrangements of gene clusters present in the specified species. Colours indicate homologous genes. Arrows indicate the direction of transcription. (**B**) Maximum-likelihood phylogenetic trees of homologous cluster genes. The trees are arbitrarily rooted using the mid-point rooting method implemented in Mega software^64^ (black triangle; see Methods). Only bootstrap support values higher than 75% are shown. Genes belonging to Mae1 and Mae2 groups are indicated. Gene names or gene-tags are indicated in brackets when paralogous genes were included in the analysis.

Previous phylogenetic analysis of *maeK* and *maeR* genes showed that the *maeK* and *maeR* homologs, that are present exclusively in some species of *Lactobacillales*, shared a common origin and are related to *maeK* and *maeR* genes present in some species of bacilli^18^. In addition, the same gene organization is found in all gene clusters of *Lactobacillales* belonging to this group which consist of *maeK* and *maeR* and, in the opposite orientation, *maeP* and *maeE* (Fig. 2A). As shown above, the analysis of the structure of MaeK revealed a distinctive feature that is shared by all MaeK belonging to this group: a degenerated CA domain. For clarity of exposition, we will name the group of gene clusters encompassing *maeK* homologs with a degenerated CA domain as the Mae1 group. In *Lactobacillales* another group of homologous *maeK* and *maeR* genes can be found (Mae2). These genes are arranged in a cluster together with a gene encoding a putative D-lactate dehydrogenase and a putative L-malate transporter (Fig. 2B) homologous to the *mleT* gene found in the malolactic cluster of *L. casei* ^19^. Contrarily to Mae1 group, HKs belonging to Mae2 contain a canonical CA ATP-binding domain (Fig. 1B).

In order to gain insight into the evolution of the *mae* gene cluster we analysed the phylogenetic relationships of *maeK*, *maeR*, *maeP* and *maeE* in a group of species selected on the basis of our previous analysis^18^. The phylogenetic reconstructions showed that genes belonging to the Mae1 group constitute well-supported groups (Fig. 2B) excepting the *maeR* tree where the group can still be identified but with low support. Nevertheless, it must be taken into account that the information content of the *maeR* dataset estimated in number of fully resolved quartets by likelihood mapping was lower than those of the other three genes analysed (79% fully resolved quartets for *maeR*, 87.25% for *maeK*, 91.99% for *maeP* and 92.16% for *maeE*). Furthermore, we tested the congruency of the topologies of the *maeR* and *maeK* phylogenetic trees attending to the tests implemented in Treepuzzle^27^. This analysis showed that for the *maeR* dataset, the topology of the *maeR* tree was not significantly better than the topology of the *maeK* tree (Supplementary Table 1). In contrast, for the *maeK* dataset, the topology of the *maeK* tree was significantly better than the topology of the *maeR* tree. This result indicates that the topology of the *maeR* tree is possibly identical to the topology of the *maeK* tree but due to the low information content of the *maeR* dataset, it could not be retrieved by the phylogenetic reconstruction procedure. In summary, the phylogenetic analyses indicate that *maeR* and *maeK* homologous genes included in this analysis have possibly evolved as a single unit.

The analyses also indicate that Mae2 group genes evolved independently of Mae1 group. First, the gene content differs between the two groups (Fig. 2A). Second, the phylogenetic analyses show that *maeK* and *maeR* genes of Mae1 and Mae2 groups are distantly related (Fig. 2B). Third, Mae2 group MaeK homologs conserve the CA domain. In this sense, it is worth noting that *Carnobacterium maltaromaticum* LMA28 harbors both gene clusters.

The close relationship between *mae* genes found in some *Bacilli* and those of the Mae1 group of *Lactobacillales* may indicate a common origin of the gene clusters found in both groups. Our results do not allow ascertaining this point. Notwithstanding, our results strongly suggest that the *Lactobacillales mae* gene clusters of the Mae1 group originated from a common ancestor cluster that was rearranged in *Lactobacillales* and has been disseminated as a single unit. This notion is further substantiated by the presence of *maeP* or *maeE* paralogs in some strains of the Mae1 group (Fig. 2B). In all cases, these paralogs are distantly related to their cognate Mae1 group genes.

### *L. casei* MaeR shows low in vitro phosphorylation levels in the presence of acetyl phosphate

**Figure 3:**
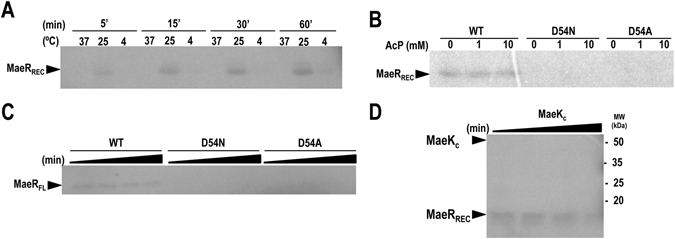
MaeR phosphorylation by acetylP and dephosphoarylation by MaeK. (**A**) Phosphorylation of the REC domain of MaeR in the presence of the radiolabeled phosphodonor acetylP and different temperatures (5, 25 and 37 °C) during different incubation times (5, 15, 30 and 60 min). (**B**) Phoshorylation of the MaeR REC domain for wild-type and mutant forms D54A and D54N with radiolabeled acetylP at room temperature for 1 h in the presence of 1 and 10 mM of cold acetylP. (**C**) Phosphorylation of MaeR full-length protein wild-type and mutant forms with radiolabeled acetylP at different incubation times (5, 10, 20 and 40 min). (**D**) Incubation of phosphorylated REC domain of MaeR in the presence of radiolabeled acetylP for 1 h and subsequent incubation with MaeK_C_ during 1, 2.5, 5 and 15 min. Full-length autoradiographies are shown in Supplementary Fig. 11

Since MaeK is a non-canonical HK lacking a functional CA domain, we checked the architecture and functionality of MaeR. *In silico* modeling with I-TASSER server^24^ showed that MaeR presents the typical domain architecture of a RR, consisting of a N-terminal phosphoacceptor REC domain (residues 1–122) and a C-terminal DNA-binding domain (type winged HTH; residues 123–229), with high structural homology to the RR PrrA from *Mycobacterium tuberculosis* that belongs to the OmpR/PhoB family (Supplementary Fig. 3). Analysis of this structural model showed that the REC domain of MaeR contains the conserved catalytic residues of RRs, including the phosphoacceptor Asp (D54) in a correct spatial disposition (Supplementary Fig. 3), suggesting that MaeR is a functional RR. Frequently, RRs can be phosphorylated *in vivo* and *in vitro* by small phosphodonors, such as acetyl phosphate (acetylP)^28^. Given the particularities of MaeK, we wondered if MaeR could be phosphorylated by acetylP. Therefore, phosphorylation assays of MaeR containing only the REC domain (residues 1–121; MaeR_REC_) were performed in the presence of radioactive ^32^P-acetylP at different temperatures (4, 25 and 37 °C) and times (5–60 min). Low phosphorylation levels were observed in MaeR_REC_ after incubation at 25 °C and at different time points (Fig. 3A). Unexpectedly, lower phosphorylation levels were observed at 37 °C, probably indicating a reduced stability of MaeR_REC_~P at high temperatures. To assess that MaeR was phosphorylated at D54, two mutant MaeR_REC_ variants at the phosphorylatable Asp (D54A and D54N) were obtained. Assays with radiolabeled acetylP using increasing concentrations of cold acetylP (1 and 10 mM) showed that the mutants were not phosphorylated in any condition assayed (Fig. 3B), confirming that the low, but consistent, level of phosphorylation of the wild-type MaeR_REC_ occurred at the phosphoacceptor Asp residue. Further phosphorylation assays using full-length MaeR (MaeR_FL_), as well as mutants D54A and D54N of this full-length protein, confirmed that the low level of phosphorylation was independent of the absence or presence of the DNA-binding domain (Fig. 3C).

Usually, phosphorylation of a RR drives its oligomerization, mainly dimerization, yielding the active conformation of the protein^29^. Even though the level of phosphorylation of MaeR was low; we sought to observe the oligomeric (dimeric) phosphorylated form by size-exclusion chromatography using MaeR_FL_ and MaeR_REC_. Size-exclusion chromatography with wild-type and mutants D54A and D54N showed that MaeR eluted from the column with a retention volume corresponding to the monomeric species in the absence or presence of acetylP (Supplementary Fig. 4). These results opened the possibility that phosphorylation did not induce oligomerization on MaeR, as it has been described for some RRs lacking DNA-binding domain, or that the low level of the phosphorylation, and consequently of the dimer, precluded the detection of this oligomeric form.

As MaeK was defective in *in vitro* autophosphorylation activity but contained the E/DxxT/N motif present in HKs with the capacity to dephosphorylate RR~P, MaeK phosphatase activity towards phosphorylated MaeR was assayed. Phosphatase assays were performed by incubating wild-type MaeR_REC_~P with MaeK_C_. In this experiment dephosphorylation of MaeR_REC_~P was not observed in the course of the reaction (Fig. 3D), supporting that MaeK_C_ does not show phosphatase activity towards MaeR~P under our assay conditions. In addition, no new band corresponding to MaeK_C_~P (Fig. 3D) was observed, suggesting that this protein cannot be phosphorylated by acetylP, which was present in the reaction. Similar results for MaeK_C_ phosphorylation were obtained when phosphoenolpyruvate was used as small phosphodonor (data not shown). Further native-PAGE experiments indicated that the MaeK_C_-MaeR_REC_ complex showed low stability or that the complex was transient (Supplementary Fig. 4), suggesting that the full-length membrane-embedded MaeK or additional partners might be needed for a productive interaction. Therefore, lack of phosphatase activity could be intrinsic to MaeK or be related to the low stability showed by the MaeK-MaeR complex.

### *L. casei* MaeR_REC_ dimerizes in a swapped conformation

**Figure 4:**
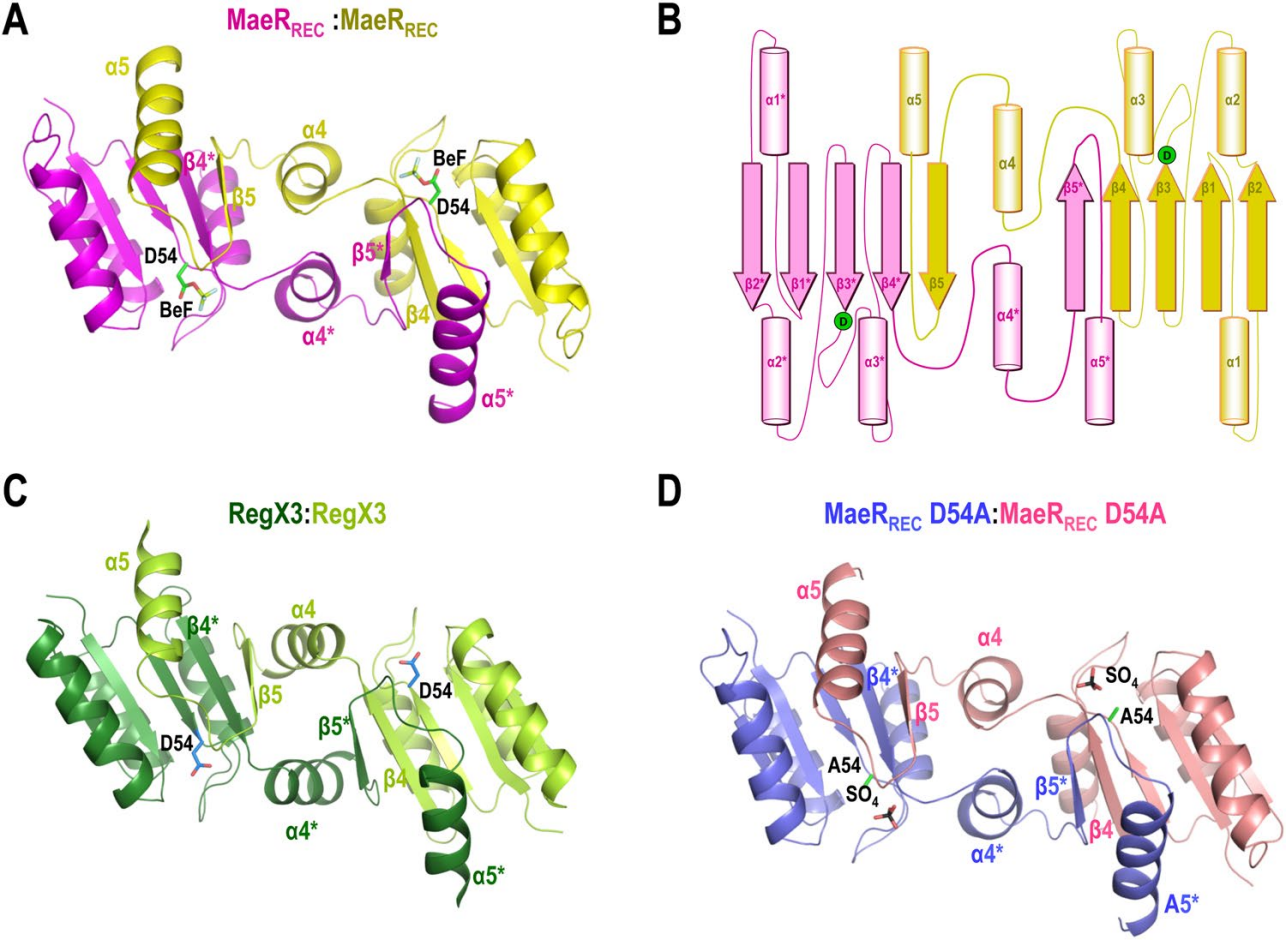
Crystal structure of MaeR_REC_ wild-type and D54A mutant. (**A**) Cartoon representation of the dimeric MaeR_REC_ wild-type structure highlighting the structural elements involved in the swapped dimerization surface (one monomer in magenta, the other in yellow) as well as the catalytic Asp bound to BeF_3_^−^ (in green). (**B**) Schematic representation of the MaeR dimer showing the structural elements involved in the swapped dimerization. (**C**) Cartoon representation of RegX3 REC domain (PDB: 2OQR) which presents the same swapped dimerization architecture as MaeR. (**D**) Cartoon representation of the dimeric MaeR_REC_-D54A structure that shows the same swapped dimer organization (one monomer in blue, the other in pink) as MaeR. The Ala residue replacing the phosphoacceptor Asp and the sulfate ion occupying the active site are highlighted in sticks.

To gain insight into the molecular basis of the activation mechanism of MaeR, we performed the structural characterization of MaeR_REC_, either in the phosphorylated or unphosphorylated conformation. To induce the phosphorylated conformation we used the phosphomimetic beryllium trifluoride (BeF_3_^−^), that is known to bind the phosphorylatable Asp leading to a structure that mimics the phosphorylated species and that has been broadly used with several RRs to this end^30, 31^. Crystals were only obtained for MaeR_REC_ in the presence of BeF_3_^−^, suggesting that this form (the phosphorylated REC domain) could represent the most compact and stable conformation. Crystals diffracted to 2.1 Å (Table 1) and the asymmetric unit contained two monomers of MaeR_REC_. Remarkably, these two monomers were organized as a dimer but by swapping some structural elements in a conformation just observed, so far, in the activated conformation of the full-length structure of the RR RegX3 from *Mycobacterium tuberculosis*, which contains the REC and DNA binding domains (RegX3_FL_; PDB:2OQR)^32^ (Fig. 4). Although the presence of this type of conformation in other RR supports its biological significance, we drove our attention to crystal packing in the MaeR structure. The solvent content of the unit cell was remarkably high (~67%), leaving a considerable free space for the dimeric quaternary structure observed in the crystal. This fact supports that packing forces do not induce the swapped conformation (Supplementary Fig. 5). The dimerization interface involves α4β5α5 structural elements, the main dimerization surface exploited by canonical RRs, such as the OmpR/PhoB family^29^, but in MaeR β5 and α5 are swapped between subunits (Fig. 3A,B and Supplementary Fig. 6). This swapped conformation is due to a motion of the C-terminal β5α5 structural elements that swap from one monomer to the other by a rotation of about 178° (calculated by Dyndom^33^), using the loop β4-α4 and α4 as a hinge (Supplementary Fig. 7). To accomplish this swapping, α4 is reoriented and placed perpendicular to α3 instead of parallel, as is observed in the OmpR/PhoB family of RR (Supplementary Figs 6 and 8). This motion allows the interaction of α4 from one subunit with α4 from the other subunit (α4*) and promotes new interactions that involve mainly β4 with β5* (and β4* with β5) (Fig. 4a and Supplementary Fig. 9). Studies of conformational dynamics in RegX3 point to the α4β5 region as critical to acquire the swapped conformation^34^. Although this swapped conformation exploited the α4β5α5 elements to generate the dimer, the relative disposition of the monomers in the dimer is completely different to that observed in the prototypical OmpR/PhoB family. Contrary to OmpR/PhoB dimers, where both active centres are placed in the same face of the dimer, they are located in opposite faces of the dimer in MaeR (Supplementary Fig. 6). The swapped MaeR_REC_ dimer displays an interface area of about 2018 Å^2^ and a solvation free energy ∆^i^G of about −34 kcal.mol^−1^, suggesting that it is a tight dimer. The fact that MaeR_REC_ presents the same type of dimerization by domain swapping as RegX3_FL_ (Fig. 4A and C), supports its biological relevance and reveals that the capacity of swapping seems to be intrinsic to the REC domain, since it is driven independently of the presence of the DNA binding domain. Furthermore, the identical structural organization of MaeR and RegX3, two phylogenetically distant RRs (MaeR belongs to the Cit family and RegX3 to the OmpR family of RRs) from unrelated microorganisms, support that domain swapping may represent a new paradigm of activation for a specific group of RRs.

**Table 1:** Crystallographic data and refinement statistics of MaeR.

Processed data	MaeR wild type	MaeR-D54A
ESRF Beamline		I29
DLS beamline	I04	
Wavelength (Å)	0.97624	0.97627
Resolution (Å)	27.91–2.11 (2.22–2.11)	43.85–3.10 (3.27–3.10)
Rmerge (%)	4.4 (67.4)	6.1 (54.0)
Rpim (%)	1.7 (26.3)	4.1 (37.3)
Mean I/δ(I)	22.1 (3.2)	11.2 (2.2)
N° reflections (observed/unique)	179137/23899 (25645/3477)	23722/7772 (3367/1119)
Completeness (%)	99.8 (98.9)	99.2 (99.1)
Redundancy	7.5 (7.4)	3.1 (3.0)
Space group	I4_1_	I4_1_
Cell dimensions (Å)	a = b = 75.79 c = 147.55	a = b = 76.64 c = 149.26
Angles (°)	α = β = γ = 90	α = β = γ = 90
**Refined data**		
Rcrys (%)	20.3	22.6
Rfree (%)	23.1	28.6
*N° atoms*		
Protein	1984	1964
Ligand/íon	10	10
Water	86	8
*B-factors* (*Å* ^*2*^)		
Protein	54.2	116.8
Ligand/ion	37.8	95.6
Water	57.3	76.7
*r.m.s deviations*		
Bond lengths (Å)	0.007	0.008
Bond angles (°)	1.27	1.29
*Ramachandran* (*%*)		
Favoured	97.06	95.42
Allowed	2.94	4.58
Disallowed region	0.0	0.0
PDB code	5LWK	5LWL

**Figure 5:**
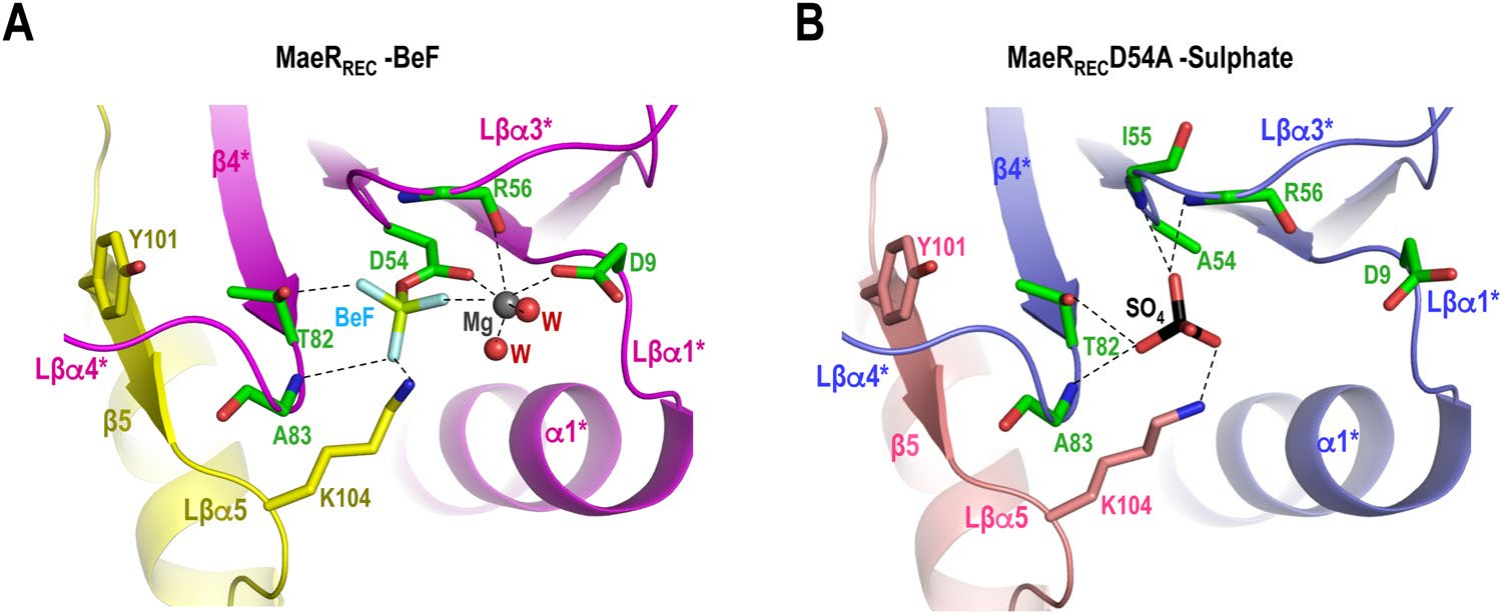
Active site organization of MaeR_REC_ wild-type and D54A mutant. (**A**) Cartoon representation of the active site in the REC domain of wild-type MaeR structure showing as sticks the catalytic residues as well as the BeF_3_^−^ (carbons in green for the magenta coloured monomer and in yellow for the yellow coloured monomer; nitrogen, oxygen, beryllium and fluor in blue, red, pale-green and cyan, respectively). The magnesium ion (in grey) and the water molecules (in red) that stabilize the active site are also shown. (**B**) Cartoon representation as in A of the active site in the mutant D54A with the presence of a sulphate ion in the active site. Catalytic residues are shown as sticks (carbons in green for the blue coloured monomer and in pink for the pink coloured monomer; sulfur in back).

A close view of the active site of each monomer shows the presence of BeF_3_^−^ bound to the catalytic Asp54 (Fig. 5A). The binding of BeF_3_^−^ to MaeR is identical to that observed in complexes of this phosphomimetic with prototypical RRs^35^. In this way, BeF_3_^−^ contacts with the conserved catalytic Lys104 in the loop connecting β5-α5 (Lβα5), Thr82 at the end of β4, the main-chain amide of Ala83 in loop connecting β4-α4 (Lβα4) and to a magnesium ion. Furthermore, the magnesium ion shows the characteristic interactions with the phosphoacceptor Asp54, the conserved Asp9, the main chain carbonyl oxygen of Arg56 and two water molecules, which are coordinated by the conserved Asp9 and Lys104, and one fluoride from BeF_3_^−^ (Fig. 5A). The residues Thr82 and Tyr101, which correspond to the Y-T or aromatic switch, are oriented towards (inward) the active site. The Y-T switch is the general mechanism for intermolecular signalling in RR and the inward conformation is adopted upon Asp phosphorylation, as it is observed in other structures of RRs phosphorylated or bound to BeF_3_^−^^36^. Interestingly, Lys104 and Tyr101, which contribute to the active site and to the Y-T switch respectively, are provided from swapped elements of the second monomer (Fig. 5A). In this way, the phosphorylated state cannot be reached unless the REC domain is swapped. Therefore, MaeR should adopt the swapped conformation to be competent for phosphorylation. Similarly, the swapped conformation has been proposed to represent the active (phosphorylated) conformation of RegX3^32^.

### *L. casei* MaeR_REC_ D54A also dimerizes in a swapped conformation

In an attempt to determine the MaeR unphosphorylated monomeric conformation, we performed crystallization studies of a MaeR_REC_ mutant form where D54 was replaced by an alanine (MaeR_REC_-D54A). MaeR_REC_-D54A crystallized in the same space group and similar size dimension than the wild-type MaeR_REC_ (Table 1). The determination of the structure of MaeR_REC_-D54A by molecular replacement revealed the presence of a swapped dimer into the unit cell identical to the one obtained for the wild type MaeR_REC_ (RMSD is 0.52 Å for 242 residues) (Fig. 4A and D). It was surprising that this non-phosphorylatable form could adopt the phosphorylated conformation but a close view of the active centre revealed the presence of a sulphate ion that was occupying the same position than the BeF_3_^−^ ion in the wild-type structure (Fig. 5). The crystals were obtained at high concentrations of sulfate (1.6 M) and it is well known that sulphates can mimic phosphate ions^37^. Indeed, this sulphate established interactions with the conserved catalytic residues Lys104, Thr82 and the main chain amide groups of Ala83 and Arg56 as BeF_3_^−^ did in the wild-type structure (Fig. 5). No interactions with the conserved Asp9 were observed due to the absence of a magnesium ion in the active site. The residues Thr82 and Tyr101 forming the Y-T switch present the inward conformation as in the MaeR_REC_-BeF structure, confirming an activated state for MaeR_REC_-D54A (Fig. 5B). The fact that the non-phosphorylatable mutant also adopts a dimeric swapped conformation indicates that this conformation is not induced upon phosphorylation. On the contrary, it supports that the swapping by itself generates the conformation competent for phosphorylation where the active site displays an organization able to accept the phosphoryl group. This notion is further supported by the coordination of a sulphate ion in the absence of a phosphorylatable Asp in the MaeR_REC_-D54A crystals.

### Asp54 participates in MaeR activation and its phosphorylation enhances binding to the *mae* promoter

**Figure 6:**
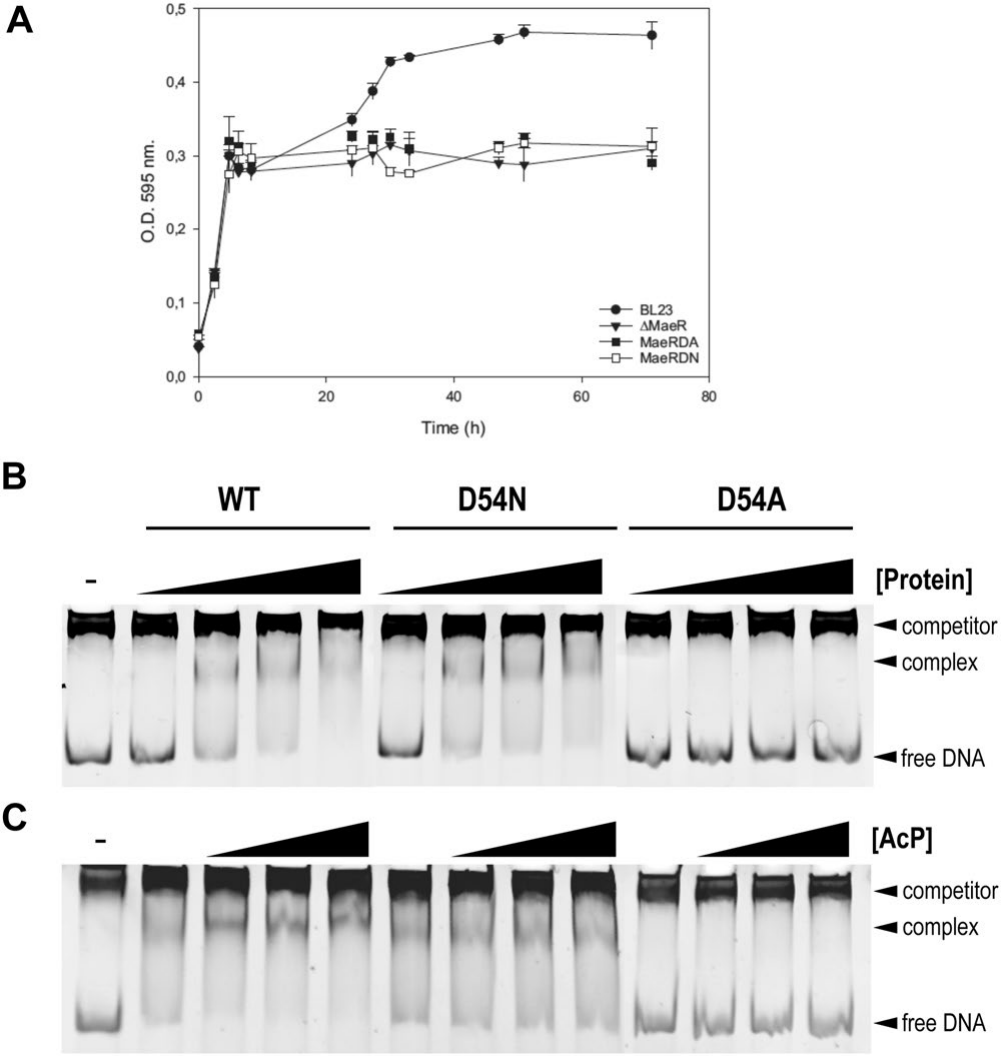
Effect of MaeR mutants on growth and DNA binding. (**A**) Growth of wild-type *L. casei* and derivative strains expressing D54A (MaeRDA) and D54N (MaeRDN) MaeR mutants in MEI medium supplemented with 5 g liter^−1^ of L-malic acid. Values represent the means of three independent experiments; error bars represent standard deviations. (**B**) Binding of MaeR and its derived mutants in Asp54 to the *mae* promoter region. Reactions were carried out in 15 μl of buffer containing 100 ng of the target DNA (*mae* promoter) and 62, 125, 250 and 375 ng of protein; (−) indicates no protein addition. (**C**) Effect of acetylP on MaeR binding. The *mae* promoter fragment was incubated with 125 ng of protein and increasing concentrations of acetylP (0, 1, 5 and 10 mM). Full-length gels are shown in Supplementary Fig. 11

Previous genetic evidence indicated that MaeR acts as a transcriptional activator of the expression of *mae* catabolic genes in *L. casei* ^18^. In order to test *in vivo* whether MaeR activity was regulated via phosphorylation of Asp54, two mutant *L. casei* strains in which this residue was replaced by Ala (D54A) or Asn (D54N) were constructed (strains MaeRDA and MaeRDN, respectively). These two mutant strains lost the capacity to grow on L-malate, (Fig. 6A) confirming the importance of Asp54, presumably through its phosphorylation, for MaeR activity *in vivo*.

We also studied the *in vitro* binding activity of MaeR_FL_ and its derivative D54A and D54N variants to the intergenic region containing the *mae* promoter as it was previously reported^18^ (Fig. 6B). Despite the low *in vitro* phosphorylation observed for MaeR with acetylP, the presence of this compound enhanced the binding activity of the wild-type protein to the *mae* promoter region (Fig. 6C). This enhancement was not observed when other possible phosphoryl donors such as ATP or phosphoenolpyruvate were used in binding reactions (data not shown). EMSA assays showed that the D54A mutant had completely lost the capacity to bind the *mae* promoter. On the contrary, binding of the D54N variant was still observed, although, as expected, its binding was not enhanced in the presence of acetylP (Fig. 6C).

### The three direct repeats in the *mae* promoter region are involved in *L. casei* MaeR recognition

**Figure 7:**
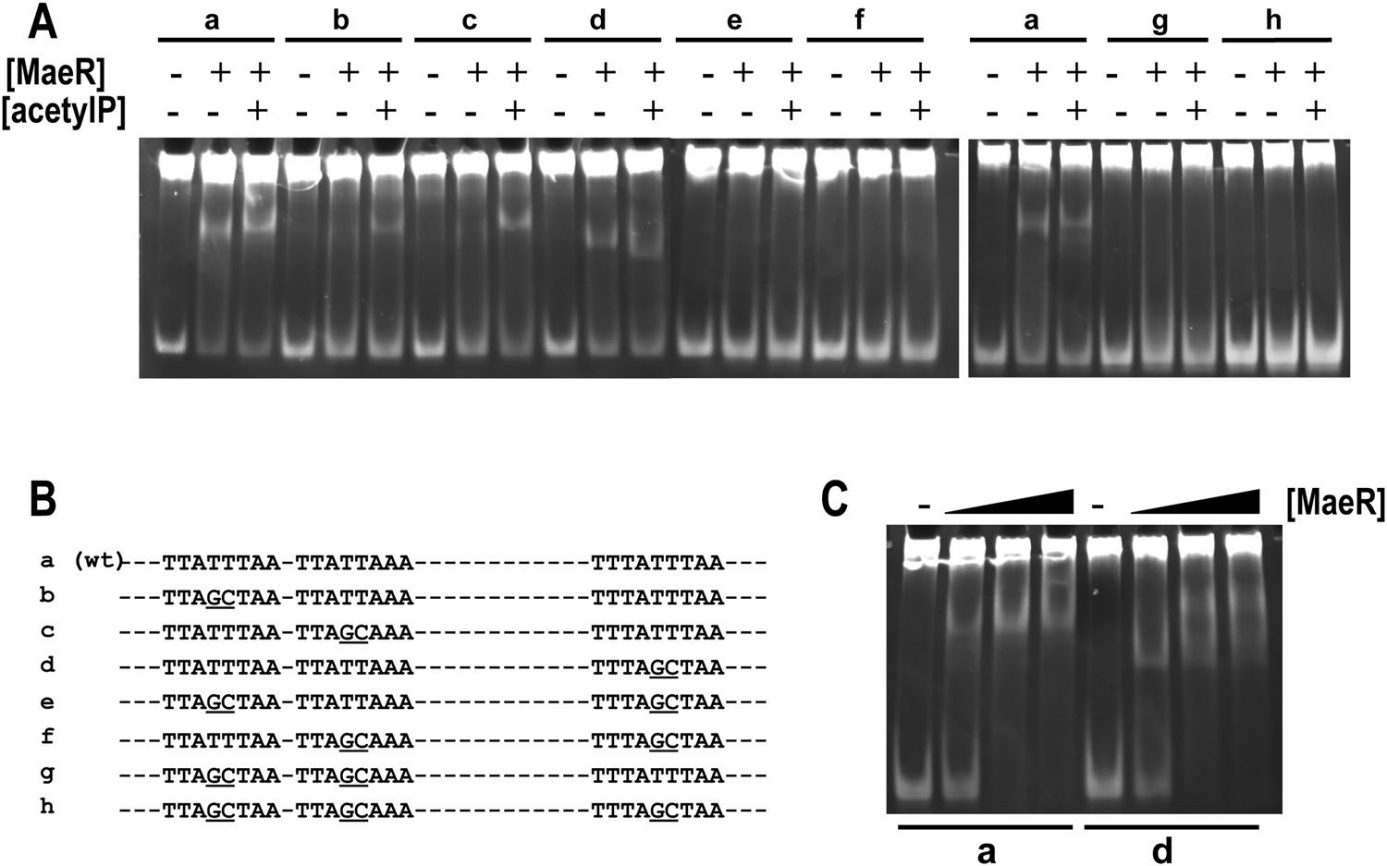
The effect of mutations in the direct repeats of the *mae* promoter on MaeR binding. (**A**) Different *mae* promoter fragments (80 bp; a, wild-type promoter; b to h, mutant forms shown in (**B**) were incubated with MaeR (125 ng) in the presence (+) or absence (−) of 10 mM acetylP. (**C**) Binding of MaeR to a fragment of the *mae* promoter: wild-type (fragment a) and a mutant in the third repeated sequence (fragment d).125, 250 and 375 ng of MaeR were added and the binding was performed in the presence of 10 mM acetylP. Full-length gels are shown in Supplementary Fig. 11

MaeR binding to the *mae* promoter protects a DNA stretch that contains three tandem repeats of the consensus sequence 5′-TTATT(A/T)AA-3′^18^. Similar repeats are present in the *mae* promoters of other LAB carrying homologous *mae* clusters, although differences in their spacing are found^18^. In order to study the unusual binding of a RR to three repeats in a promoter region, we performed mutations of the central TT pair in any of the two first sites, which are separated by 1 bp. These mutations resulted in diminished MaeR binding activity, which was mostly detected in the presence of acetylP (Fig. 7A and B). Specifically, compared to the wild-type sequence, mutation of two (or the three) sites resulted in a complete loss of MaeR binding (Fig. 7A). However, the mutation of just the distal third site did not produce a drastic effect in binding, although the pattern of DNA retardation changed (Fig. 7B and C). These results confirmed that phosphorylation of MaeR increased DNA binding affinity and that the studied DNA repetitions were the target for MaeR recognition. Albeit, they suggested a major contribution of the two first sites in MaeR recognition.

## Discussion

Structural and phylogenetic analyses show that *L. casei* MaeKR proteins belong to the citrate family of TCS, as well as the characterized DcuSR and CitAB TCS. These systems are usually involved in the regulation of the utilization of dicarboxylic and tricarboxylic acids^18, 38–43^. Notwithstanding, there are remarkable differences between the Mae1 group and other previously characterized TCS, most notably, the presence of a degenerated CA domain in the MaeK-like sensor proteins, which precludes MaeK ATP-dependent autophosphorylation. Furthermore, in characterized sensor kinases of this family, a cognate transporter usually functions as a co-sensor^23^, but apparently this is not the case for MaeK since the absence of L-malate transporters did not affect the induction of *mae* genes^19^.

The phylogenetic analyses carried out here and in a previous study^18^ have shown that Mae1 group homologs are most closely related to cognate TCS of *Bacillaceae* involved in the regulation of L-malate utilization. The analyses also indicate that the *mae* gene clusters present in Lactobacillales proceed from an ancestral cluster that was assembled or rearranged in *Lactobacillales*. The phylogenetic relationships between the genes of this group and the gene arrangement are conserved thus indicating that the Mae1 gene cluster has evolved as a unit. This observation also suggests that the peculiarities of the Mae1 TCS evolved as an adaptation to the genetic and physiological environment of an ancestor of *Lactobacillales* and has been conserved during the evolutionary history of this gene cluster. Therefore, the mechanism of signal transduction is possibly identical in all Mae1 TCS.

MaeK and MaeR exert their function by activating the expression of L-malate catabolic genes (*maeP* and *maeE*) and the expression of their own genes upon the presence of L-malate in the medium^18, 19^. Replacement of the phosphorylatable Asp residue in MaeR resulted in the loss of the ability to grow on L-malate of the corresponding *L. casei* mutant strains. Furthermore, phosphorylation of MaeR with acetylP, albeit low, enhanced MaeR DNA-binding activity *in vitro*, which suggests that MaeR~P has higher affinity for its cognate DNA binding sites than unphosphorylated MaeR. EMSA experiments also showed that the D54N MaeR mutant was not sensitive to acetylP (Fig. 6C). Taken together, these results strongly suggest that phosphorylation of MaeR at Asp 54 is required to function *in vivo*.

The structure of the MaeR REC domain has provided some clues on the phosphorylation pathway of this RR. The structure revealed that dimerization of MaeR involves the swapping of structural elements between the REC domains of the MaeR monomers. The analysis of the structural constraints indicated that the swapped form of MaeR is competent for accepting a phosphate at Asp 54. Since MaeR was predominantly present as a monomer *in vitro*, the low number of MaeR dimers would explain the low level of MaeR~P observed during phosphorylation assays. The possibility to catch in the crystal structure this reduced pool of swapped MaeR molecules is explained by the dynamics of the crystallization process. Although the equilibrium between the monomer and the swapped dimer is displaced toward the monomer in solution, the crystallization conditions, including the presence of BeF_3_^−^, can stabilize the swapped form, reaching enough concentration to nucleate. Once this solid nucleus has been formed, it would work as a protein sink shifting the equilibrium in the solution towards the swapped form that is added to the solid crystal. The fact that identical α4β5α5-swapping is present in the activated forms of RegX3_FL_ and MaeR_REC_ supports that this type of dimerization represents the paradigm of an alternative mechanism of RR activation. We anticipate that this activation mechanism by domain swapping of α4β5α5 elements will be found in more RR. Indeed, the domain swapping has been observed in the other RRs as in the bacteriophytochrome RRs RtBRR (*Ramlibacter tataouinensis;* PDB 5IC5) and AtBRR (*Agrobacterium tumefaciens*; 5BRJ)^44^ or the master regulator for sporulation Spo0A (*Bacillus stearothermophilus*; PDB 1DZ3)^45^, although in these cases different structural elements were swapped, suggesting that variations of this mechanism could be common among RRs.

The low proportion of MaeR~P obtained via acetylP possibly accounts for the enhanced DNA-binding activity of MaeR observed in EMSA assays, but, how is MaeR phosphorylated *in vivo* and which is the role of MaeK in this process? The fact that *maeK* mutants in different species do not activate *mae* genes in response to the presence of L-malate^18, 22^ clearly indicate that MaeK is a functional L-malate sensor. However, it is intriguing how a HK devoid of its ATP-dependent kinase activity can operate in signalling. One possibility is that MaeK could promote MaeR swapping and dimerization in response to L-malate, fostering the phosphoacceptor competent conformation of this RR. A comparable situation has been proposed for DesKR TCS, which regulates genes involved in membrane fluidity in *Bacillus subtilis* ^46^. In this system, the α1α5 surface in the RR DesR is allosterically coupled to homodimerization, favouring the RR autophosphorylation. This surface is also mediating the association of the HK DesK to DesR and it participates in a process of RR activation in the absence of phosphorylation^46^. The possibility of the involvement of MaeK in allosteric regulation of MaeR is also suggested by studies on RegX3 (the other RR with an identical swapped conformation), which requires the presence of the HK SenX3 in order to be phosphorylated and to bind to its target promoters^47^. However, these examples differ from MaeK in the fact that DesK and SenX3 are functional kinases.

If MaeK was participating in a process promoting the phosphoacceptor-competent swapped conformation of MaeR, phosphoryl donors could reach and react effectively with MaeR. However, the nature of these phosphoryl donors and whether MaeK induces the swapped conformation is uncertain. In *S. pyogenes* (Mae1 group), it has been described that *ptsI* and *ptsH* mutants, defective in the Enzyme-I and HPr proteins of the phosphoenolpyruvate: sugar phosphotransferase system (PTS), are unable to induce *maePE* genes and have a consequent growth defect on L-malate^22^. The PTS is responsible for taking up carbohydrates with their concomitant phosphorylation by using phosphoenolpyruvate, a glycolytic intermediate, as a phosphoryl donor. It is conceivable that the physiological changes imposed by the lack of a functional PTS will produce changes in the concentration of intracellular metabolites, some of which may be potential phosphoryl donors (e.g. acetylP), that would result in an altered MaeR phosphorylation status. PTS components might also directly phosphorylate or affect the activity of other factor(s) (including MaeK) involved in MaeR phosphorylation. In fact, the Enzyme-I and HPr PTS components have been demonstrated to be involved in many protein regulatory processes^48^. The elucidation of these two extremes deserves further investigation in the *mae* system of *L. casei*.

Our results show that MaeKR form a TCS with a non-canonical signalling mechanism, expanding the variety of mechanisms by which HK transfer the sensed signals to RR. How a HK devoid of an ATP-binding domain performs a role in the phosphorylation of its cognate RR constitutes a new scenario in the field of TCS function. In addition, the domain swapping seems to represent a new paradigm for RR activation that waits to be extended to other TCS. Finally, why the particular characteristics of MaeKR have been developed for L-malate sensing and utilization in *Lactobacillales* and at which point and how MaeKR diverged from its relatives in the TCS citrate family are stimulating questions.

## Methods

### Bacterial strains and growth conditions

Strains used in this work are listed in Table 2. Lactobacilli were grown in MRS medium (Difco) at 37 °C under static conductions. Growth assays in the presence of L-malic acid were carried out at 30 °C in malic enzyme induction medium (MEI) as previously described^18^. Inoculation was performed with cells grown in MRS for 16 h and washed twice with 1 volume of sterile distilled water. Growth was monitored by measuring O.D. at 595 nm. At least three independent replicates of each growth curve were obtained. Results were expressed as averages and plus and minus standard deviations.

**Table 2:** Strains and plasmids used in this study.

Strain or plasmid	Characteristics or relevant genotype	Source or reference
*Escherichia coli*		
DH5α	F^−^ *endA1 hsdR17 gyrA96 thi*-*1 recA1 relA1 supE44* Δ*lacU169* (F80 *lacZ* ΔM15)	Stratagene
BL21-(DE3)-RIL	F^−^ *ompT hsdS*(*r*_*B*_^*−*^ *m*_*B*_^*−*^) *dcm* ^+^ *Tet* ^*r*^ *gal endA λ*(DE3) *Hte* [*argU ileY leuW Cam* ^*r*^]	Agilent
*Lactobacillus casei*		
BL23	wild type strain	^65^
BL315	BL23 Δ*maeR*	^18^
BL322	BL23 Δ*maeK*	^18^
MaeKΔC-t	BL23 harboring a deletion of the C-terminal domain of *maeK*	This study
MaeKΔC-t + MaeK	MaeKΔC-t harbouring plasmid pT1maeK	This study
MaeRDA	BL23 *maeR*D54A	This study
MaeRDN	BL23 *maeR*D45N	This study
*plasmids*		
pQE80	*cis*-repressed, IPTG inducible, N-terminal His_6_-tagged recombinant protein expression vector; Amp^r^	Qiagen
pQEmaeR	pQE80 with cloned *maeR* gene	^18^
pQEmaeRDA	pQE80 with cloned *maeR*D54A gene	This study
pQEmaeRDN	pQE80 with cloned *maeR*D54N gene	This study
pQEmaeRR	pQE80 with cloned receiver (REC) domain of *maeR* (amino acids 1 to 121)	This study
pQEmaeRRDA	pQEmaeRR with cloned receiver (REC) domain of *maeR*D54A (amino acids 1 to 121)	This study
pQEmaeRRDN	pQEmaeRR with cloned receiver (REC) domain of *maeR*D54N (amino acids 1 to 121)	This study
pNIC28-Bsa4	LIC vector. IPTG inducible, N-terminal His_6_-tagged recombinant protein expression; Km^r^	^66^
pNIC-MaeK_C_	pNIC28-Bsa4 with cloned cytoplasmic domain of MaeK	This study
pRV300	Insertional vector for *Lactobacillus*, Amp^r^, Ery^r^	^67^
pRVDmaeK-Ct	pRV300 containing a 2 kb with a deletion of the C-terminal domain of *maeK* cloned at SpeI and XhoI sites	This study
pRVmaeRDA	pRV300 with a 1.2 kb fragment cloned at XhoI and SacI containing *maeR* with a mutated codon 54 (GAT to GCT)	This study
pRVmaeRDN	pRV300 with a 1.2 kb fragment cloned at XhoI and SacI containing *maeR* with a mutated codon 54 (GAT to AAT)	This study
pT1maeK	pT1NX with cloned *maeK* expressed from its own promoter. Ery^r^	^18^

*E. coli* strains were grown in LB medium at 37 °C under agitation (200 rpm). When required antibiotic concentration used were 5 μg/ml erythromycin for *Lactobacillus*, 100 μg/ml ampicillin, 50 μg/ml kanamycin and 20 μg/ml chloramphenicol for *E. coli*.

### Deletion of MaeK C-terminal domain

In order to obtain a BL23 derivative strain harbouring a deletion of the C-t domain of *maeK*, flanking fragments of the region to be deleted were amplified using the primer sets MaeK-Ct-F1/MaeK-Ct-R1 and MaeK-Ct-F2/MaeK-Ct-R2, respectively (Supplementary Table 2). As *maeK* 3′-end overlaps the *maeR* 5′-end, a stop codon was introduced after the amino-acid 423, and a region upstream *maeR* encompassing the RBS was conserved (Supplementary Fig. 10). The corresponding pairs of PCR fragments were combined into one fragment by PCR, digested with SpeI/XhoI, and cloned in pRV300 to generate the plasmid pRVDmaeK-Ct. This plasmid was transformed by electroporation in *L. casei* BL23 as described previously^18^ and one erythromycin-resistant clone carrying the plasmid integrated by a single crossover was grown in MRS without erythromycin for ~200 generations, antibiotic-sensitive clones were isolated and, among them, one was selected in which a second recombination event led to the deletion of the C-terminal domain of *maeK*, as subsequently confirmed by sequencing of a PCR-amplified fragment spanning the deleted region. The construction resulted in a derivative strain (MCt) encoding a truncated MaeK protein (1–423 aa; Supplementary Fig. 10). Complementation of the deletion of the C-terminal domain of *maeK* was achieved by transforming the plasmid pT1maeK which harbours a *maeK* gene under control of its own promoter^18^ into strain MCt.

### Replacement of the MaeR phosphorylatable Asp54 residue by Ala or Asn

Two BL23 derivative strains where the phosphorylatable amino acid of MaeR (D54) was replaced by Ala or Asn were obtained. To this end, a 1.2 kb region encompassing the entire *maeR* gene was amplified by PCR with primers CitbUp and CitbDown. The PCR product was digested with XhoI and SacI and cloned into pRV300 digested with the same enzymes. This plasmid was used as a template for inverse PCR reactions with oligonucleotide pairs Mut-citb1/Mut-citb2 and Mut-citb3/Mut-citb4, respectively. The amplified products were treated with DpnI and used to transform *E. coli* DH5α. The presence of the required mutations was confirmed by sequencing and selected plasmids (pRVmaeRDA and pRVmaeRDN) were transformed into *L. casei* BL23 by electroporation. Strains with chromosomally integrated plasmids were selected by erythromycin resistance and the second recombination event leading to the replacement of wild type *maeR* by the mutated alleles (strains MDA and MDN) was achieved as described for the MaeKΔC-t strain.

### Expression plasmids

A *maeR* fragment encoding the receiver (REC) domain (1 to 122) was amplified by PCR with oligonucleotides maeRR-F/maeRR-R. The PCR product was digested with BamHI/SacI and cloned in pQE80 digested with the same enzymes, giving pQEmaeRR. Expression plasmids for purification of mutated variants of MaeR in D54 were constructed by inverse PCR with Mut-citb1/Mut-citb2 and Mut-citb3/Mut-citb4 oligonucleotide pairs as described for the integrative plasmids using pQEmaeR (expressing full-length MaeR^18^), and pQEmaeRR as templates. The cytoplasmic part of MaeK protein, MaeK_C_, were cloned in pNIC28-Bsa4 vector by ligase-independent cloning (LIC) using DNA fragments amplified with the oligonucleotide pairs MaeK_C__pNIC28/MaeK_pNIC28.

### Protein expression and purification

All proteins with the exception of MaeR full length protein and MaeR_REC_ were expressed in the BL21-codonplus(DE3)-RIL strain using 1 mM IPTG at 20 °C overnight. Proteins were purified by affinity chromatography in a 5-ml HisTrap FF column (GE Healthcare) equilibrated with buffer A (20 mM Tris-HCl pH 7.5, 20 mM (NH_4_)_2_SO_4_, 500 mM NaCl), washed with buffer A plus 30 mM of imidazol and eluted with buffer A plus 300 mM of imidazol. MaeR full length protein and the MaeR_REC_ domain protein were expressed in DH5α strain using 1 mM IPTG at 20 °C overnight. The purification of proteins was performed using 1 ml Histrap FF columns (GE Healthcare) equilibrated with buffer C (20 mM Bis-Tris pH 6, 20 mM (NH_4_)_2_SO_4_, 500 mM NaCl, 10%glycerol), washed with buffer C plus 30 mM of imidazol and eluted with buffer C plus 300 mM of imidazol. The MaeR mutants used (D54A and D54N) were expressed and purified as wild type MaeR proteins. All the proteins used for *in vitro* assays were loaded in a gel filtration column, Superdex75 16/60 (GeHealthcare) for MaeR proteins and Superdex200 16/60 (GE Healthcare) for MaeK_C_ protein. The proteins were concentrated around 10 mgml^−1^ with a 10 K Amicon Ultra Centrifuge Filters (Millipore) and stored at −80 °C.

### Protein crystallization, data acquisition and processing

Crystals of wild-type MaeR_REC_ and the D54A variant were obtained using the vapor diffusion method and using the sitting drop technique in the Crystallogenesis facility of IBV. Drops of wild-type MaeR_REC_ and the D54A variant were set up by mixing 0.4 μl of protein solution at 4.5 mgml^−1^ and 15 mgml^−1^, respectively, in buffer 50 mM Bis-Tris pH 6.0 and 200 mM ammonium sulfate with 0.4 μl of different reservoir solutions. Wild-type MaeR_REC_ crystals were obtained in 1.2 M magnesium sulfate and 0.1 M MES pH 6.5 while D54A crystals where obtained in 1 M lithium sulfate, 0.5 M ammonium sulfate and 0.1 M sodium citrate. For cryoprotection, wild type crystals were added to a solution containing the reservoir solution at 0.8 M of magnesium sulfate plus 15% ethylenglycol and 25% sucrose while D54A crystals were soaked in the same reservoir solution plus 13% ethylenglycol, 7.3% sucrose and 3% PEG 6000. Diffraction quality was tested from single crystals at 100 K on European Synchrotron Facility (ESRF, Grenoble, France), Diamond Light Source Synchrotron (DLS, (Didcot, UK) and Spanish Synchrotron Radiation Facility ALBA (ALBA, Cerdanyola del Vallès, Spain). The complete diffraction data set was collected at the ESRF in a nitrogen-gas stream at ID29 at 2.8 Å and 3.1 Å for wild type and D54A mutant, respectively. Higher resolution at 2.1 Å was obtained for wild type crystals diffracted at I04 at DLS one year later after first crystals appeared in the plate. Crystallographic data was processed and scaled with the programs MOSFLM and SCALA from the Collaborative Computational Project, Number 4, CCP4 suite^49^ or the XDS Program Package^50^. Details of the data-collection are shown in Table 1. The structure of wild-type MaeR_REC_ was determined by molecular replacement using the program MrBUMP^51^ and the structure of the D54A mutant was obtained by molecular replacement with the program Phaser^52^ using the refined structure of the wild-type protein. The final models were obtained using a combination of refinement cycles with the program Refmac5^53^ and manual building with the program Coot^54^. Details of the refinement are shown in Table 1. Structural analysis of the structures was performed using other programs such as Ncont and Superpose contained in CCP4 suite^49^. Figures were produced using PyMOL (http://www.pymol.org).

### Autophosphorylation assays

The MaeK_C_ autophosphorylation assays were performed with [γ-^32^P] ATP (3000 Ci/mmol Perkin Elmer) at concentrations of 0.5 mM of cold ATP and 0.1 and 0.5 μCi/ μl of radioactive ATP during 10 and 60 min. Samples were subjected to SDS-PAGE on 15% gel and phosphorylated proteins were visualized by phosphorimaging using a Fluoro Image Analyzer FLA-5000 (Fuji) and evaluated with the MultiGauge software (Fuji).

### Isothermal titration calorimetry

The binding affinity measurements for MgCl_2_ and AMPPNP and the indicated protein were taken with a Nano-ITC (TA Instruments). Experiments were carried out at 25 °C with 0.03 mM of protein in the cell and mixed with 0.7 or 5 mM nucleotide in the syringe by 25 injections of 2 μl each at intervals of 180s under continuous stirring. Data integration, correction and analysis were carried out using NanoAnalyze program (TA Instruments) with a single-site binding model.

### Circular Dichroism

Circular Dichroism spectroscopy (CD) experiments were performed using a JASCO J-810 spectrometer that was coupled to a Peltier temperature controller (PTC-423S). Protein solutions in 50 mM phosphate buffer, pH 7.5, were adjusted to a protein concentration of 10 μM and then placed in a round cuvette Helma with a 0.1 cm path length. Data were collected over a wavelength range of 190–250 nm in milliabsorbance units at 25 °C, averaged and background corrected using the Spectra Manager software provided with the instrument. These data were transformed to ellipticity *θ* using the relationship [*θ* = milligrades × 10lCp] where l is the path length, *C* is the concentration of the protein (M) and p is the number of peptidic bonds. The analysis was done using DICHROWEB^55^ and VADAR^56^

### Synthesis of radioactive acetylP

The radioactive [^32^P]acetylP used in the MaeR phosphorylation was obtained incubating for 2 h at room temperature 1.5U of acetate kinase with 100 μCi/ μl of [γ-^32^P] ATP (3000 Ci/mmol Perkin Elmer) in 2.5 mM Tris pH 8, 6 mM potassium acetate, 1 mM Cl_2_Mg buffer. The [^32^P]-acetylP was freed from acetate kinase by filtering using Microcon-10 kDa Centrifugal Filter Unit (GE Healthcare). The pure [^32^P]-acetylP was stored at −20 °C.

### MaeR phosphorylation assays

MaeR was phosphorylated incubating 1 mg ml^−1^ of the protein at room temperature for the indicated incubation times with 12.5 mM [^32^P]-acetylP in a solution containing 50 mM Tris-HCl pH 8.0, 500 mM KCl, 5 mM MgCl_2_, 20 mM DTT and 1 mM EDTA. The samples were subjected to SDS-PAGE on 15% gel and phosphorylated proteins were visualized by phosphorimaging using a Fluoro Image Analyzer FLA-5000 (Fuji) and evaluated with the MultiGauge software (Fuji).

### Phosphatase assays

The MaeR_REC_ protein phosphorylated for 1 h as described in the *MaeR phosphorylation assays* above, was incubated with equimolecular concentrations of MaeK_C_ at room temperature for the indicated incubation times and the samples were subjected to SDS-PAGE on 15% gel. Phosphorylated proteins were visualized by phosphorimaging using a Fluoro Image Analyzer FLA-5000 (Fuji) and evaluated with the MultiGauge software (Fuji).

### Electrophoretic mobility assays

A 219 bp fragment spanning the *maeP-maeK* intergenic region was amplified by PCR with the oligonucleotide pair FP1/FP2. Different combinations of mutations in the three putative MaeR binding sites (5′-TTATT(A/T)AA-3′ direct repeats) in which the central TT pair was replaced by GC were obtained by PCR using oligonucleotides carrying the desired mutations that amplified a 80 bp fragment. The combinations of the different oligonucleotides (see Supplementary Table S2) were: FP5/FP6 (wild type sequence), FP8mut2/FP6 (mutant at site 1), FP9mut3/FP6 (mutant at site 2), FP5/FP7mut1 (mutant at site 3), FP8mut2/FP7mut1 (mutant at sites 1 and 3), FP9mut3/FP7mut1 (mutant at sites 2 and 3), FP10mut4/FP6 (mutant at sites 1 and 2), FP10mut4/FP7mut1 (mutant at the three sites). EMSA assays using these fragments were carried out in 15 μl of 2.5 mM Tris-HCl pH 7.5, 100 mM NaCl, 25 mM MgCl_2_, 0.25 mM EDTA, 0.25 mM dithiothreitol, 10% glycerol containing 1.5 μg of salmon sperm DNA, 100 ng of each fragment and varying amounts of MaeR or its variants MaeR-D54A and MaeR-D54N (62 to 375 ng) with or without acetylP (1 to 10 mM). These mixes were incubated at 37 °C for 30 min before being resolved in 6% polyacrylamide (19:1) gels with 1X TAE (40 mM Tris-acetate pH 7.6, 1 mM EDTA) as running buffer at 100 V. The gels were stained with ethidium bromide.

### Phylogenetic analysis

Homologous sequences of *mae* genes (*maeK*, *maeR*, *maeP* and *maeE*) present in species belonging to *Lactobacillales* were identified and retrieved from complete genomes deposited at MBGD^57^. Redundant sequences were excluded from the analysis. Outgroup sequences were selected on the basis of our previous phylogenetic analysis^18^. Sequences were aligned at the T-Coffee server (http://tcoffee.crg.cat) with the M-Coffee tool and default options^58^. Gaps and low homology regions were removed using Gblocks^59^. Details on sequences used and alignments are available from the authors upon request.

The best-fit models of amino acid substitution were selected using the ProtTest 2.4 server (http://darwin.uvigo.es/software/prottest2_server.html)^60^. The Akaike information criterion (AIC) was adopted to select the best model. The models selected were used to estimate the corresponding phylogenies by maximum likelihood using the PhyML tool^61^ implemented in the T-Rex server (http://www.trex.uqam.ca)^62^. Bootstrap support values were obtained from 1000 pseudoreplicates. Phylogenetic information content in the sequence alignments was analyzed by likelihood mapping as implemented in TreePuzzle 5.3rc^63^. Congruence among topologies for *mae* gene trees was evaluated using the tests implemented in TreePuzzle 5.3rc^27^. Phylogenetic trees were visualized and arbitrarily rooted using the mid-point rooting method implemented in Mega software^64^.

## Electronic supplementary material

Supplementary Information
