# Supplementary material for: The malate sensing two-component system MaeKR is a non-canonical class of sensory complex for C4-dicarboxylates
Source: Sci Rep. 2017 Jun 2;7:2708. doi: 10.1038/s41598-017-02900-z (PMC5457438; doi:10.1038/s41598-017-02900-z)
Supplement: Supplementary file 1 — Supplementary Information [file 41598_2017_2900_MOESM1_ESM.pdf]

The malate sensing two-component system MaeKR is a non-canonical class of sensory complex for C4-dicarboxylates

Miguel-Romero L, Casino P, Landete JM, Monedero V, Zúñiga M and Marina A.

Table of Contents: Page

Section	Page
Supplementary Tables	2
Supplementary Figures	3-13

Supplementary Table 1. Comparison of tree topologies.

Dataset	Tree	p-1sKH[a]	p-SH[b]	c-ELW[c]	2sKH[d]
maeR	maeR	1.000 +	1.000 +	0.835 +	Best +
	maeK	0.167 +[e]	0.173 +	0.165 +	+
maeK	maeR	0.001 -[e]	0.002 -	0.000 -	-
	maeK	1.000 +	1.000 +	1.000 +	Best

[a] One sided Kishino-Hasegawa test based on pairwise Shimodaira-Hasegawa tests.

[b] Shimodaira-Hasegawa test.

[c] Expected Likelihood Weight.

[d] Two sided Kishino-Hasegawa test.

[e] Plus signs denote the confidence sets. Minus signs denote significant exclusion. All tests used 5% significance level.

Supplementary Table 2. Primers used in this study

Primer	Sequence
MaeK-Ct-F1	TTTTACTAGTTGACTGCCTTGCGCGGTCAT
MaeK-Ct-R1	TTGAGCCTCCTCGTGGTAGTCC**TCA**TAATGTTAACAGCGTTTTGGTT
MaeK-Ct-F2	AACCAAAACGCTGTTAACATTA**TGA**GGACTACCACGAGGAGGCTCAA
MaeK-Ct-R2	TTTTCTCGAGTAGGAGAAACGGTGCCAATG
Mut-citb1	TACTT**GCT**ATCCGCCTCAAAGACGGCAAC
Mut-citb2	CGGAT**AGC**AAGTAAAACAAGTTGGATG
Mut-citb3	TACTT**AAT**ATCCGCCTCAAAGACGGCAAC
Mut-citB4	CGGAT**ATT**AAGTAAAACAAGTTGGATG
CitbUp	TTTTCTCGAGACAATATGACGAATTGGCCATCTACT
CitbDown	TTTTGAGCTCTTACGACAGCGCATGTGGTGCCG
FP1	AGCCCCATAGCCGCCATCC
FP2	CGTTGATTTGTTGGGTGTAACC
FP5	GCTTCATCAGGGGAAGATTA
FP6	CATCTTGTGAATAGTAAAACAATCG
FP7mut1	CATCTTGTGAATAGTAAAACAATCGCTTA**GC**TAAATGGG
FP8mut2	GCTTCATCAGGGGAAGATTA**GC**TAACTTATTAAAC
FP9mut3	GCTTCATCAGGGGAAGATTATTTAACTTA**GC**AAACCATAAAC
FP10mut4	GCTTCATCAGGGGAAGATTA**GC**TAACTTA**GC**AAACCATAAAC
MaeRR-F	TTTTGGATCCGACGATGACGATAAGATGACGAACATCCTAATCGTTG
MaeRR-R	TTTTGAGCTCCTTGGTTCTGTAACGTTGAATACTTT
$MaeK_C$ _pNIC28	TACTTCCAATCCATGAAAGAAATTGCTAGGCTGCTTG
MaeK_pNIC28	ATCCACCTTTACTGTTATCAACGATTAGGATGTTCGTC

Underlined sequences correspond to restriction sites introduced for cloning. The mutated positions are in bold.

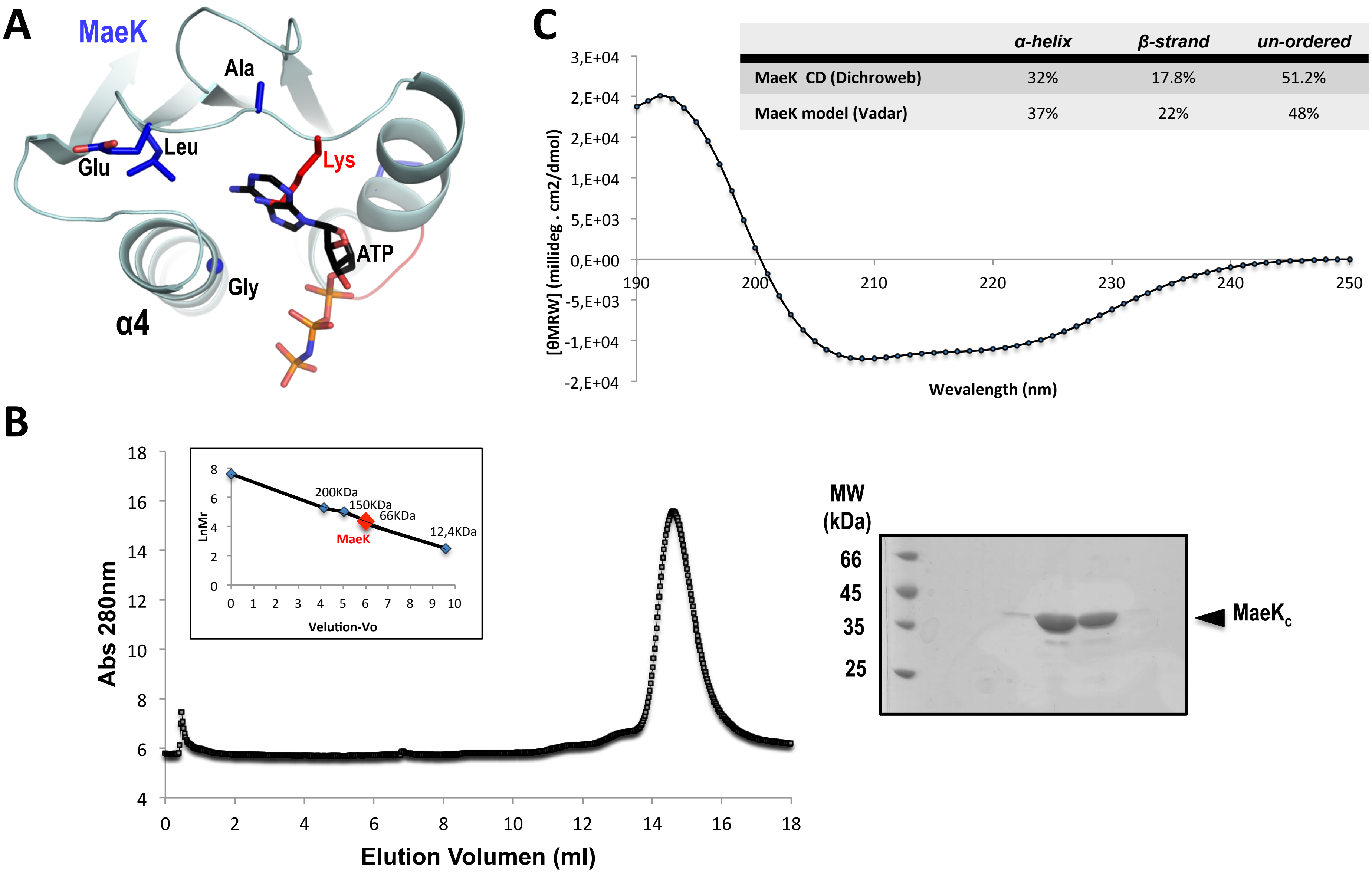

	α-helix	β-strand	un-ordered
MaeK CD (Dichroweb)	32%	17.8%	51.2%
MaeK model (Vadar)	37%	22%	48%

Supplementary Fig. 1. A) Cartoon representation of the modeled degenerated CA domain of MaeK obtained with I-TASSER. Superposition with the CA of EnvZ bound to AMPPNP (ATP analog; PDB 4KP4) shows a probable clash between a Lys and the nucleotide. B) Size exclusion chromatography assays of $MaeK_C$ using Superdex200 (30/100) column. SDS-PAGE shows purity of $MaeK_C$ after SEC. C) Circular dichroism spectra for the complete cytoplasmic portion of MaeK, $MaeK_C$. Secondary structure composition is obtained from the analysis of the CD spectra using the program Dichroweb and from *in silico* protein modeling using the program Vadar.

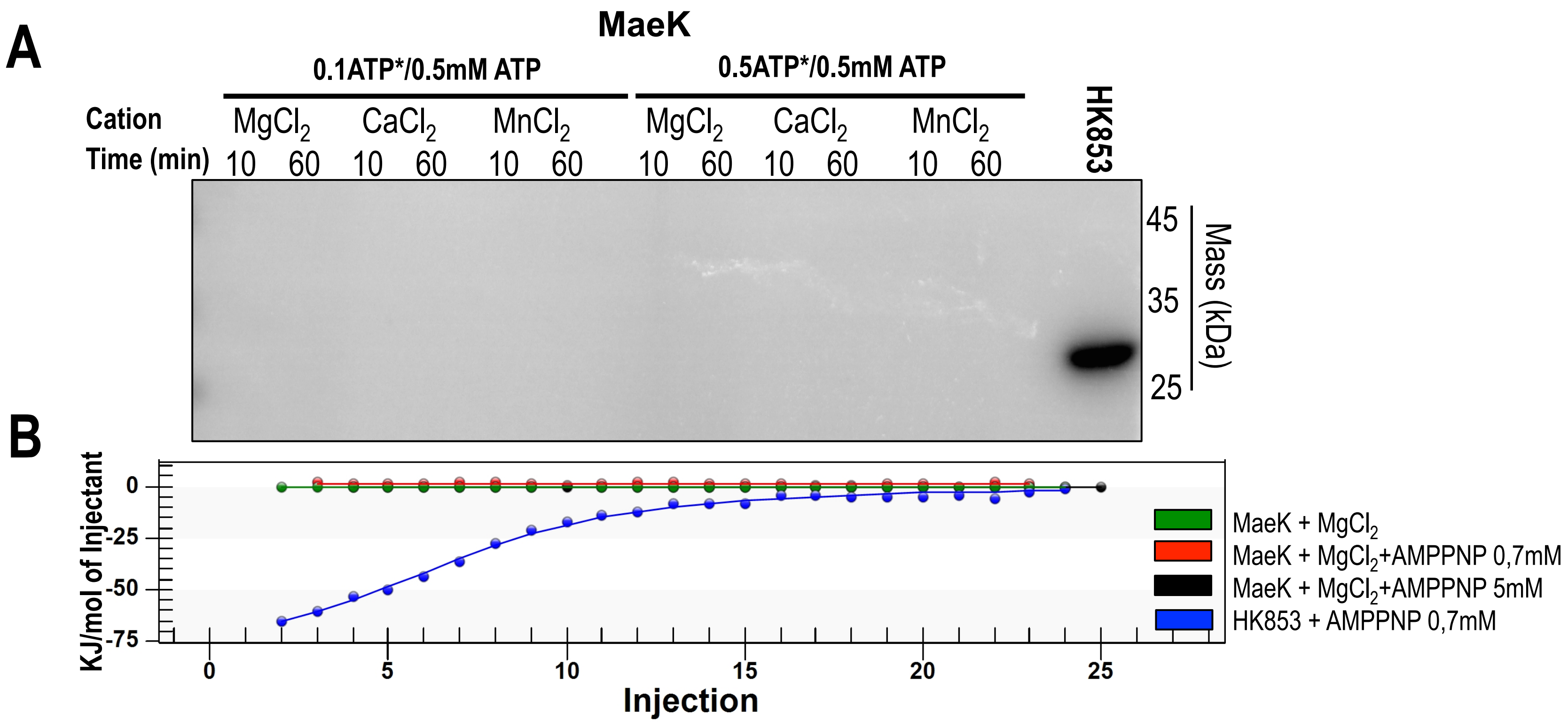

	n	ΔH	ΔS	Kd(M)
MaeK - $MgCl_2$	10	-0,728	54,99	>1xE^{-3}
MaeK - $MgCl_2$+AMPPNP 0,7mM	10	-10,26	23,02	>1xE^{-3}
MaeK – $MgCl_2$+AMPPNP 5mM	10	2,671	66,39	>1xE^{-3}
HK853 - AMPPNP 0,7mM	1,37	-84,17	-185,8	9,135xE^{-6}

Supplementary Fig. 2. A) Autophosphorylation assay for $MaeK_c$, visualized by autorradiography, upon incubation with radiolabeled [γ-^{32}P]ATP (ATP*) for 10 and 60 min in the presence of different divalent cations and ATP*/A TP (cold ATP) ratios. As control experiments, the autophosphorylation of HK853 in one of this conditions (10 min, $MgCl_2$ and 0.1 ATP*/ 0.5 ATP) is shown. Similar levels of autophosphorylation of HK853 were observed in the rest of conditions. B) Binding affinity determination by ITC of MaeK for $MgCl_2$ and the non-hydrolyzable ATP analog, AMPPNP. Heat responses per injection are plotted with solid lines to fit one-site binding model. Obtained Kd (μM) constants are shown in the table below. Binding affinity determination was also performed for HK853 and AMPPNP as a positive control for binding.

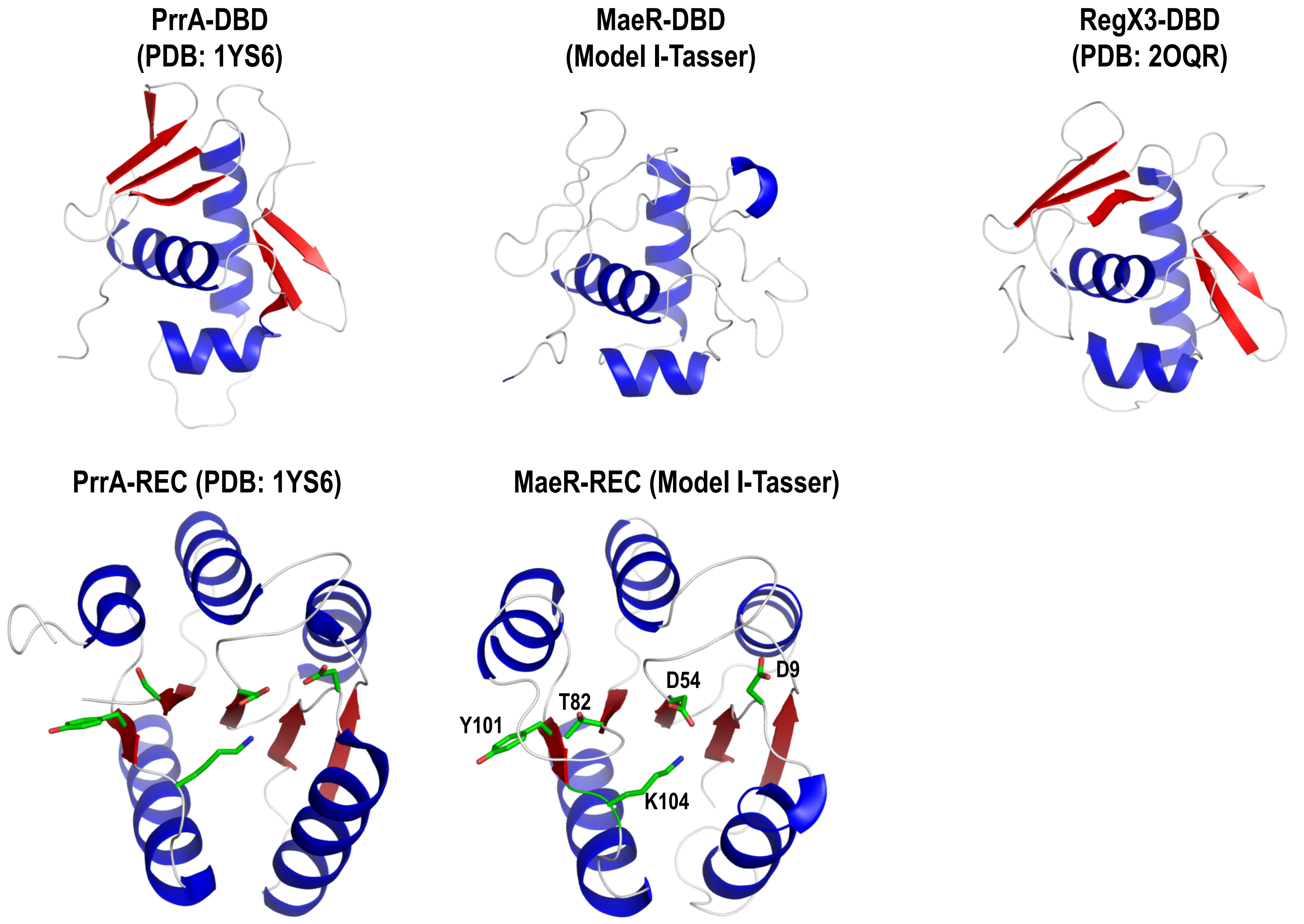

Supplementary Fig. 3. Cartoon representation of modeled MaeR using I-TASSER. The modeled DBD and REC are compared to PrrA, a response regulator with close structural homology. The DBD of RegX3 is also showed for comparison. Secondary structure is Highlighted in blue for α helices and red for β strands.

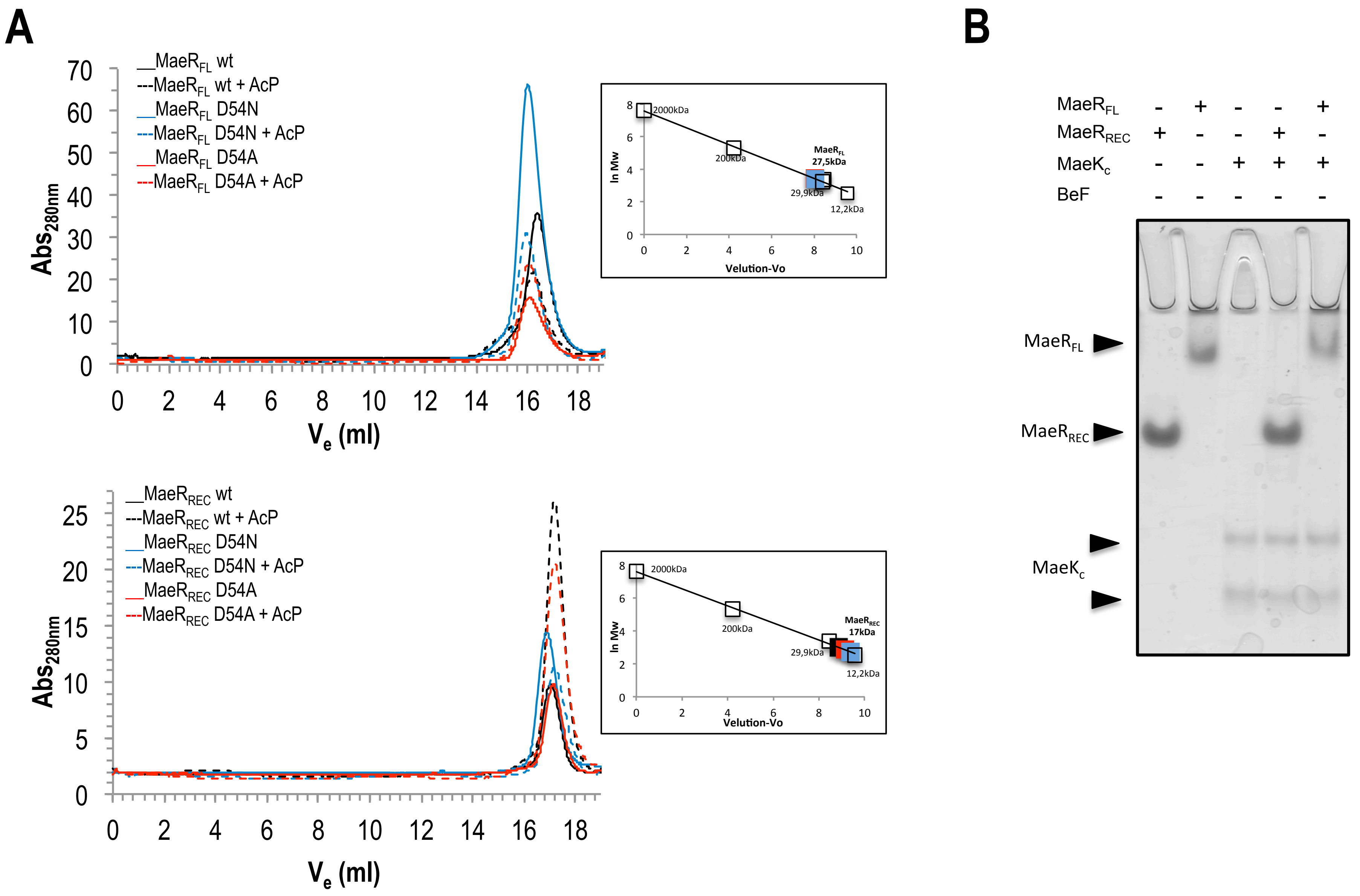

Supplementary Fig. 4. A) Size exclusion chromatography assays for MaeR$_{REC}$ and MaeR$_{FL}$ proteins either wild-type and mutant forms (D54A and D54N) in the absence and presence of acetylP using a Superdex 75 (30/100). B) Native gel electrophoresis of MaeR$_{FL}$ or MaeR$_{REC}$ alone and incubated with MaeK complete cytoplasmic portion, MaeK$_C$.

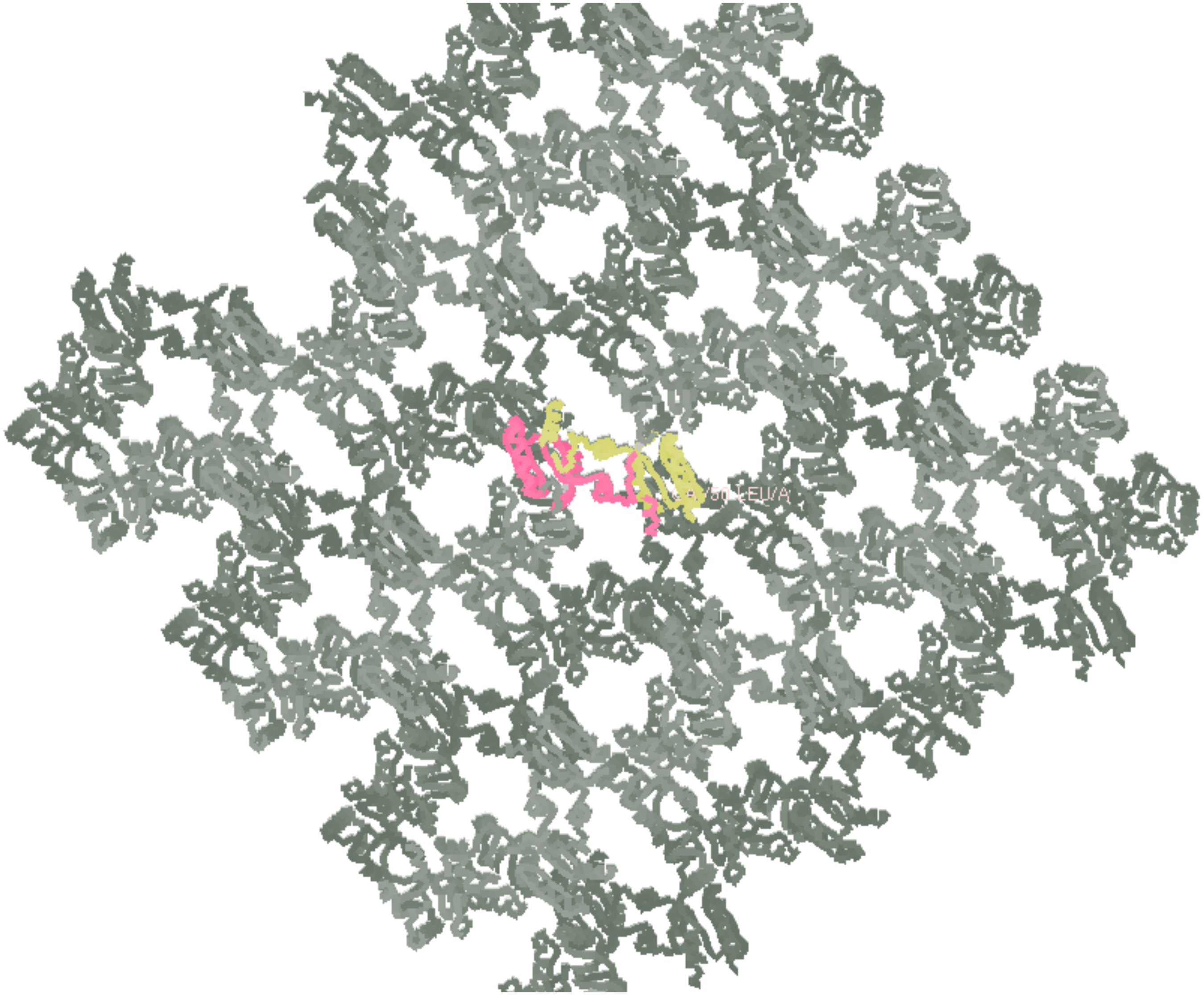

Supplementary Fig. 5. Crystal packing of $MaeR_{REC}$

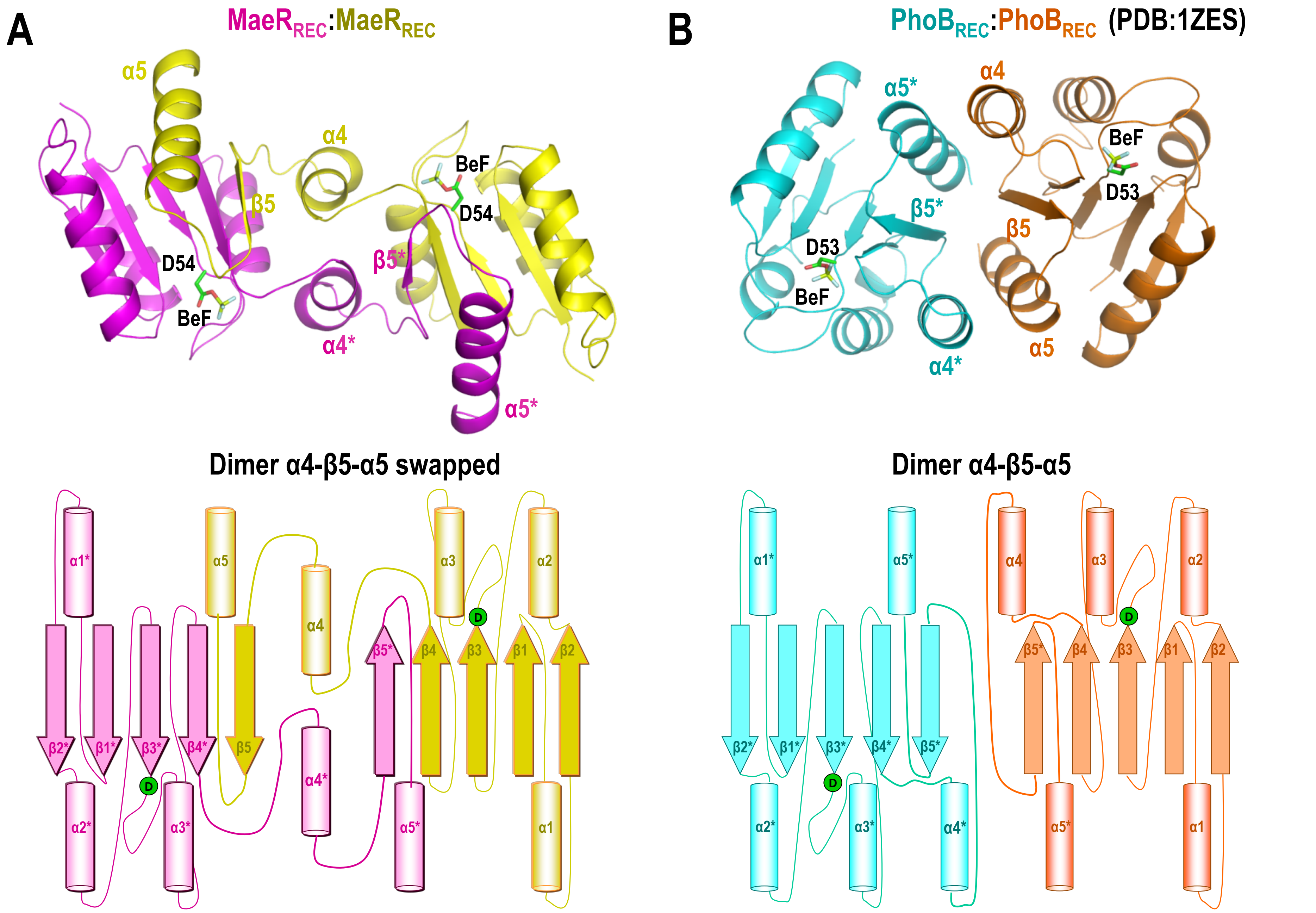

Supplementary Fig. 6. A) Cartoon representation of MaeR$_{REC}$ dimer structure. Below a scheme representation of a swapped dimer α4-β5-α5. B) Cartoon representation of PhoB$_{REC}$ and a scheme representation of a α4-β5-α5 dimer.

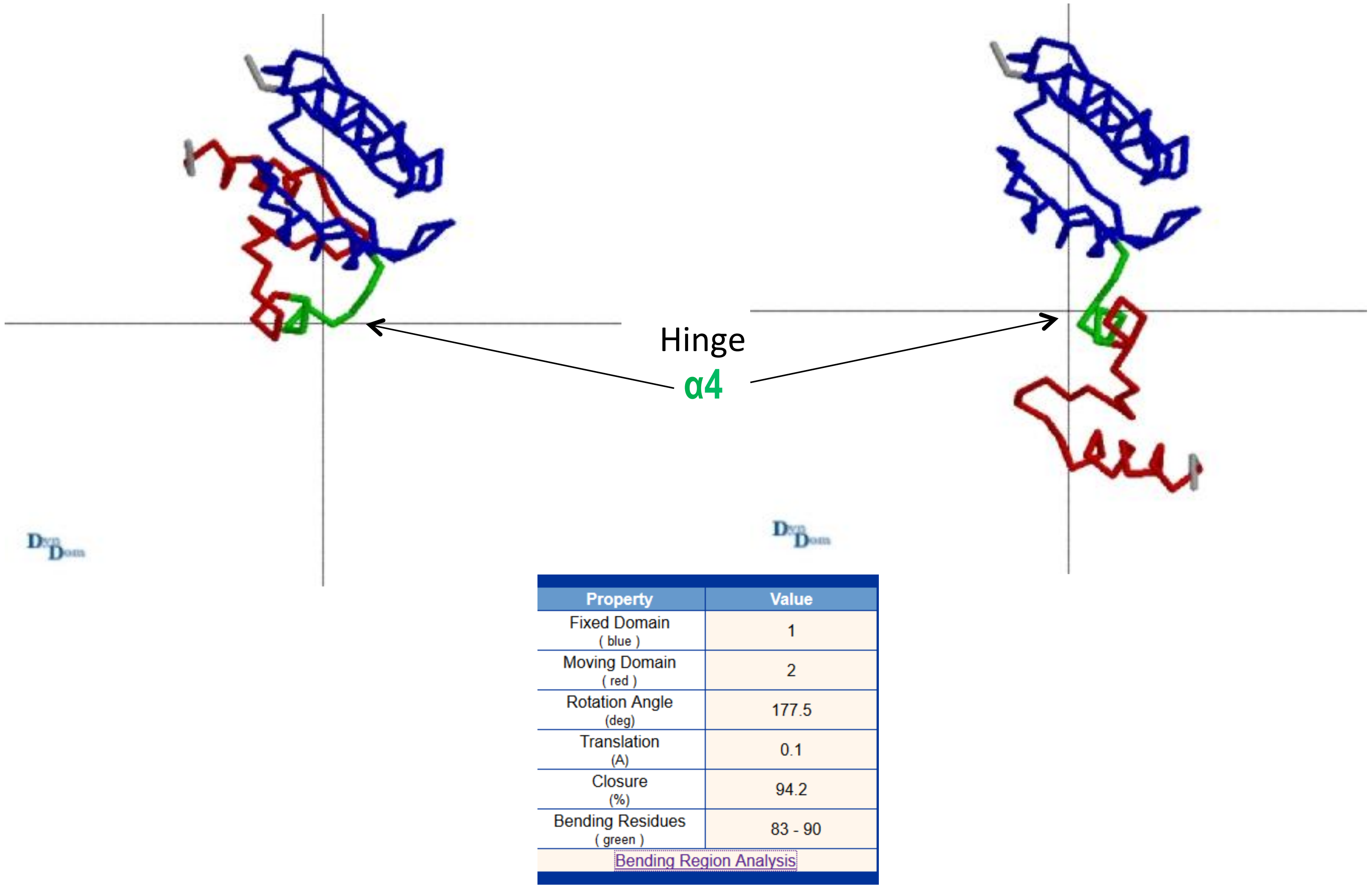

Property	Value
Fixed Domain (blue)	1
Moving Domain (red)	2
Rotation Angle (deg)	177.5
Translation (A)	0.1
Closure (%)	94.2
Bending Residues (green)	83 - 90
Bending Region Analysis	

Supplementary Fig. 7. Dyndom calculation for the movement of a monomeric $MaeR_{REC}$ in order to adopt a α4-β5-α5 swapped conformation .

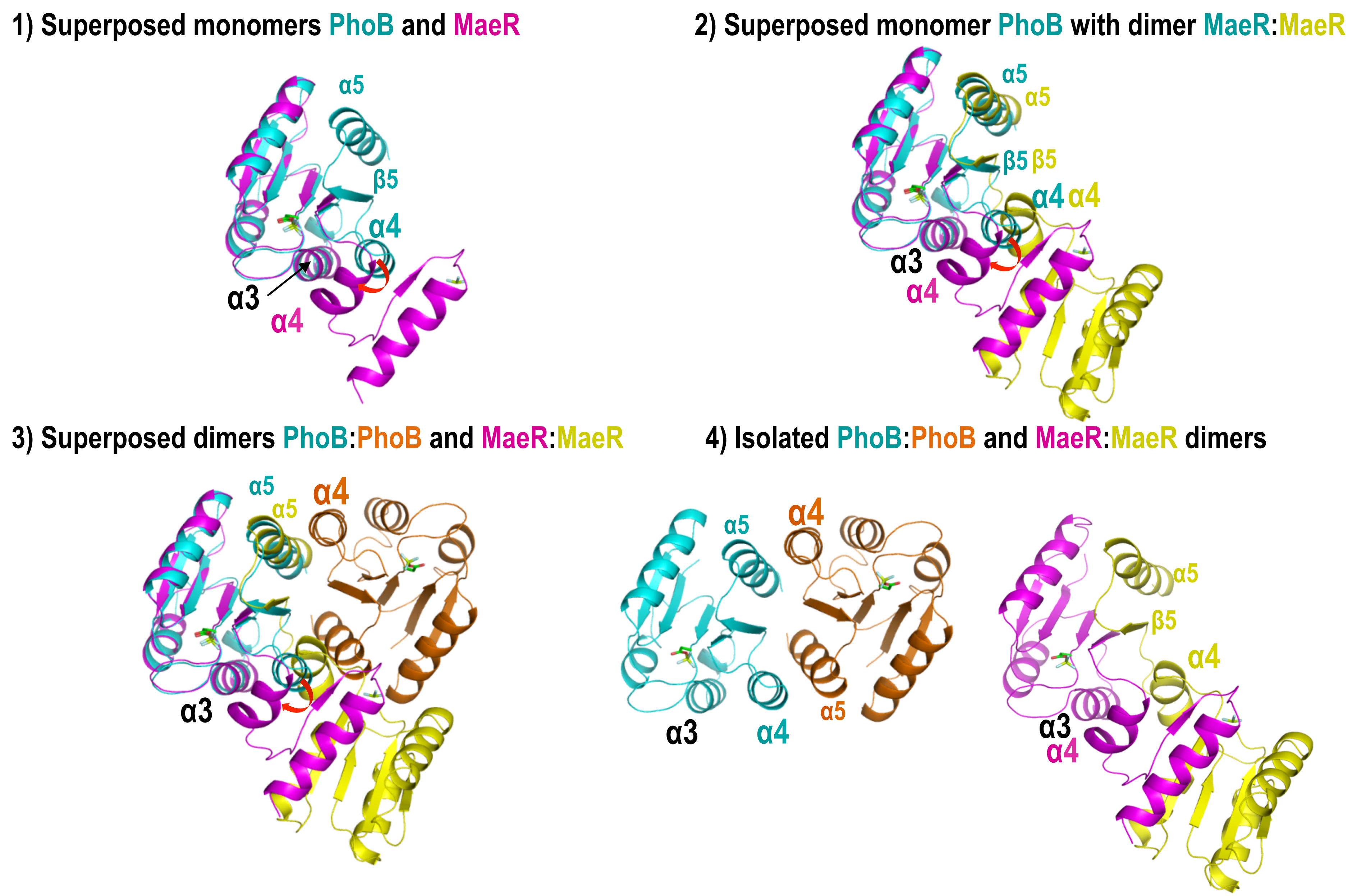

Supplementary Fig. 8. Superposition of PhoB and MaeR REC domains highlighting in several steps the displacement of α4 which allows the shift from a non swapped to a swapped dimer α4-β5-α5.

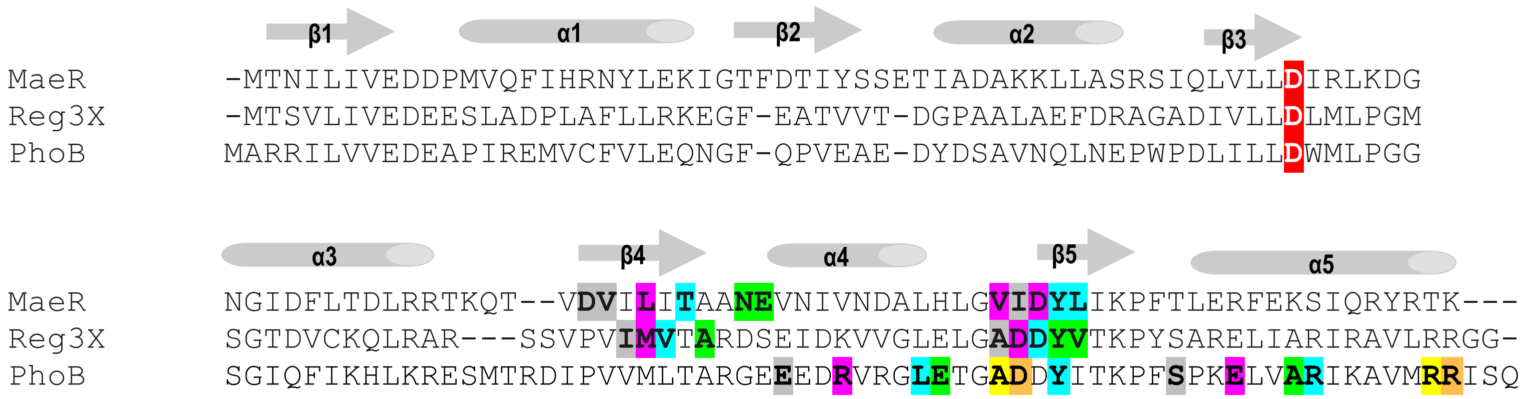

Supplementary Fig. 9. Sequence and structural alignment of the REC domain of MaeR with RegX3 and PhoB. The phosphorytable Asp are highlighted in red. The residues highlighted in gray, magenta, cyan, green, yellow, orange and ligthpink are those involve in the dimerization interface. For each sequence, identical color means that these residues interact with each other.

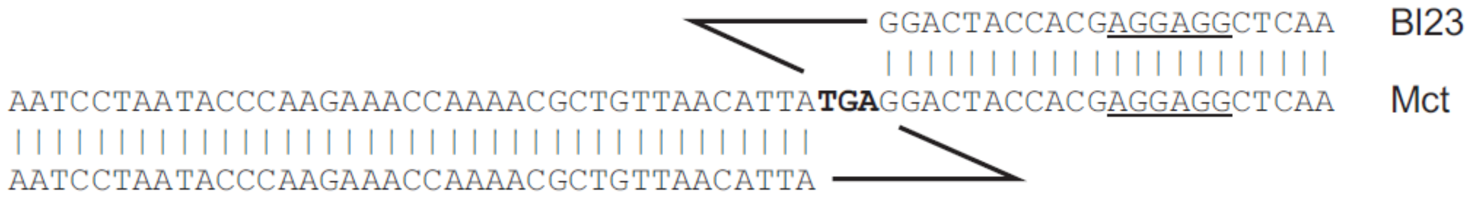

Supplementary Fig. 10. Sequence comparison of the MCt derivative strain with the parental strain BL23. The in-frame translational stop codon introduced in MCt strain is indicated in bold characters. The ribosome binding site upstream maeR is underlined. The zigzagged line represents intervening sequence.

Figure 3 panel A

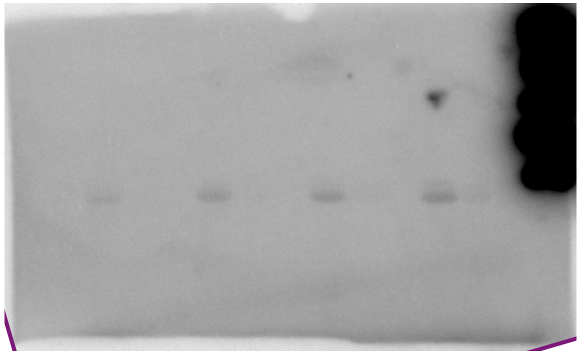

Figure 3 panel B

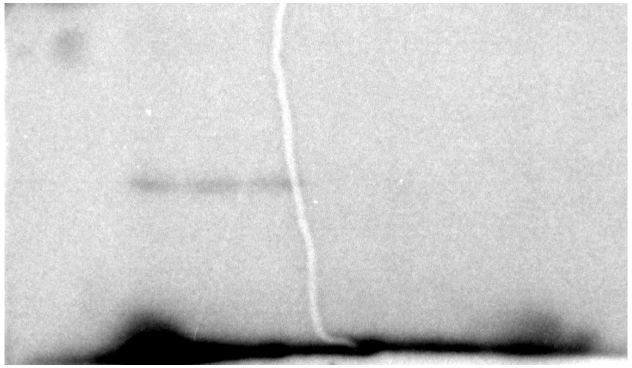

Figure 3 panel C

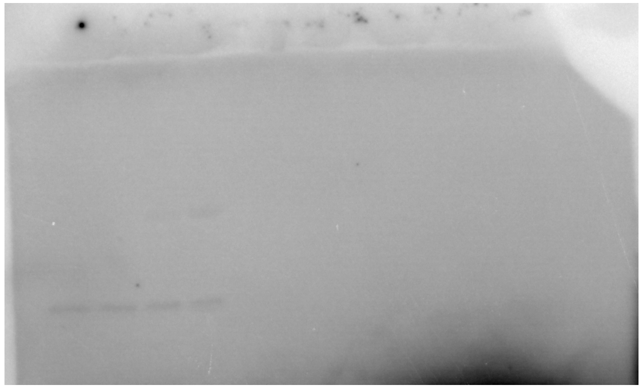

Figure 3 panel D

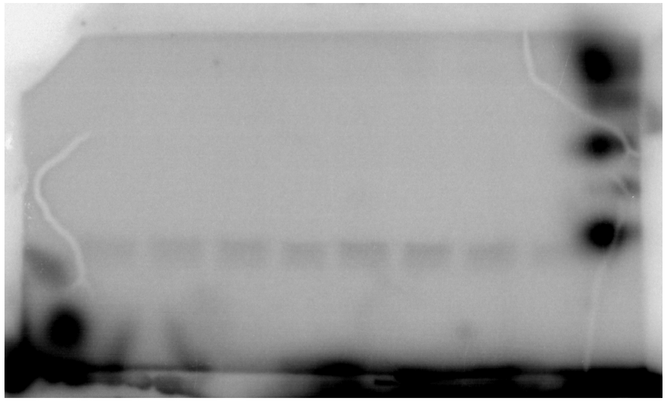

Supplementary Fig. 11-*Cont.* Full-length autoradiographies and gels from Figure 3, Figure 6 and Figure 7.

Figure 6

Panel B

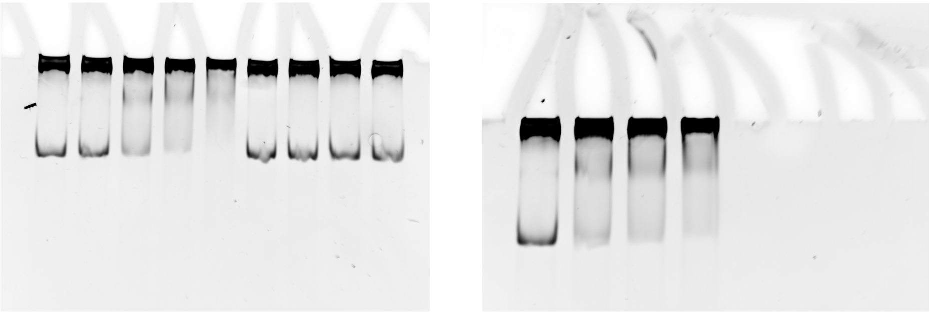

Panel C

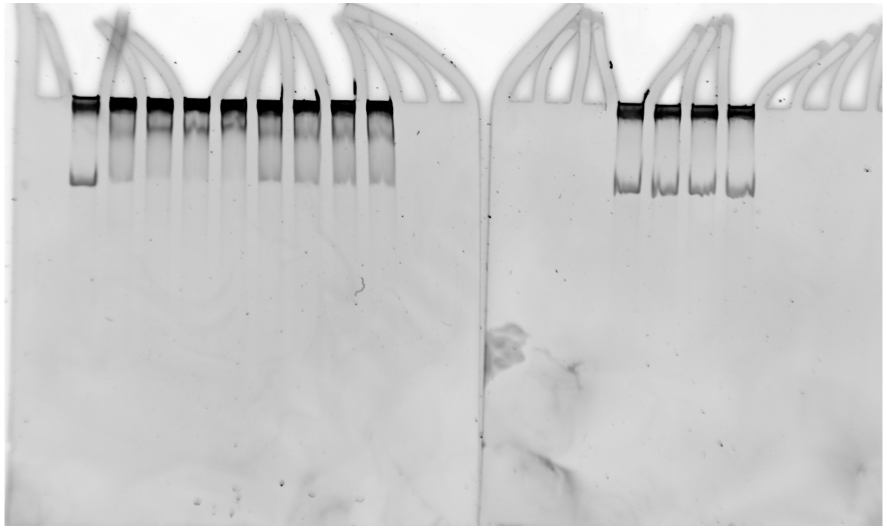

Figure 7

Panel A

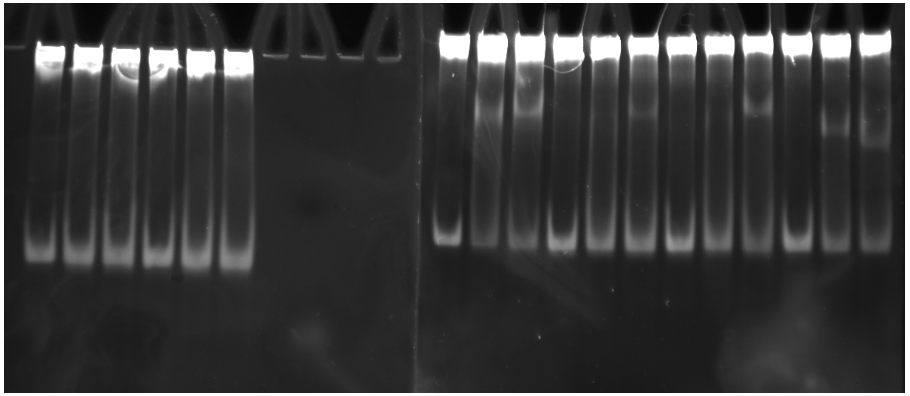

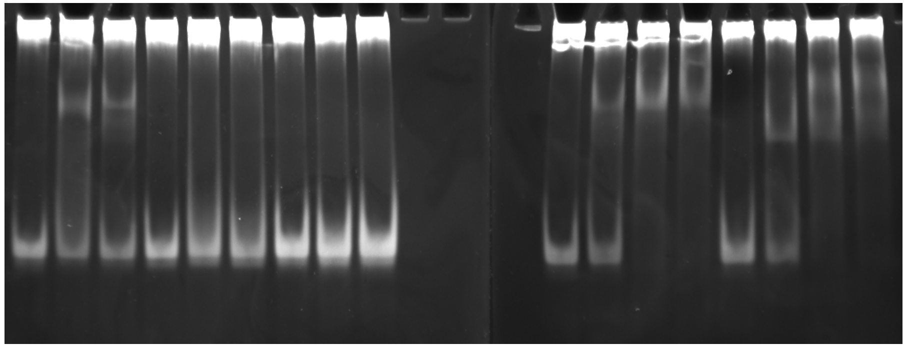

Panel A **Panel C**

Supplementary Fig. 11-*Cont.* Full-length autoradiographies and gels from Figure 3, Figure 6 and Figure 7.
